# A Review on Recently Developed Antibacterial Composites of Inorganic Nanoparticles and Non-Hydrogel Polymers for Biomedical Applications

**DOI:** 10.3390/nano14211753

**Published:** 2024-10-31

**Authors:** Anastasiia V. Shabalina, Valeriy A. Kozlov, Ivan A. Popov, Sergey V. Gudkov

**Affiliations:** 1 Prokhorov General Physics Institute, Russian Academy of Sciences, 119333 Moscow, Russias_makariy@rambler.ru (S.V.G.); 2 Institute of Biology and Biomedicine, Lobachevsky State University of Nizhny Novgorod, 603950 Nizhny Novgorod, Russia

**Keywords:** composite, antibacterial, polymer, nanoparticles, biomedical application

## Abstract

Development of new antibacterial materials for solving biomedical problems is an extremely important and very urgent task. This review aims to summarize recent articles (from the last five and mostly the last three years) on the nanoparticle/polymer composites for biomedical applications. Articles on polymeric nanoparticles (NPs) and hydrogel-based systems were not reviewed, since we focused our attention mostly on the composites of polymeric matrix with at least one inorganic filler in the form of NPs. The fields of application of newly developed antibacterial NPs/polymer composites are described, along with their composition and synthetic approaches that allow researchers to succeed in preparing effective composite materials for medical and healthcare purposes.

## 1. Introduction

Over the last few centuries, the development of civilization has led to global environmental problems that human society has only recently begun to recognize. Currently, new safe and cost-efficient methods and industrial approaches, resource-extracting processes, and human everyday life are gradually being developed and implemented. However, environmental deterioration already affects the quality of life and humans’ health. In the biomedical sector, bacterial infections are still an important opened issue. Acquired antibiotic resistance of the bacteria makes medical devices (either disposable or long-term used, such as implants), hygiene items, and even presence in a medical institution unsafe and potentially dangerous. That is why the development of new antibacterial materials for solving biomedical problems has become an extremely important and very urgent task.

**Figure 1:**
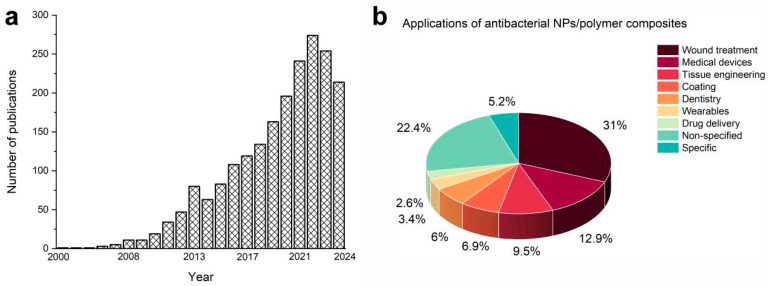
(**a**) Histogram indicating the publication activity in the field (according to the PubMed). (**b**) Pie diagram showing the application of the polymer/NPs composites in medical fields.

The development of composites containing polymer and nanoparticles (NPs) for biomedical purposes is a fast-growing field with a number of diverse applications. According to the PubMed source, overall, about 1800 articles with the key words “composite AND polymer AND nanoparticles AND antibacterial” have been published to-date (see Figure 1a). The first publication was found to be made in 2000, the next one was published in 2001, after which a slow rise can be noticed, with the first doubling of the number of articles published per year observed in 2008. Afterward, a gradual and steady increase in the number of published articles can be seen until now (see Figure 1a). Thus, new and improved composites of NPs and polymers are constantly being developed, and this field deserves close attention from researchers worldwide [1,2].

The present review aims to summarize recent articles (from the last five, but mainly from the last three years) on the NPs/polymer composites prepared for biomedical applications. We excluded from the overview the articles dedicated to polymeric NPs and hydrogel-based systems, focusing our attention mostly on the composites of polymeric matrix with at least one inorganic filler in the form of NPs (metal, oxide, etc.). The fields of application of newly developed antibacterial NPs/polymer composites are described, along with their composition and synthetic approaches that allow researchers to succeed in preparing effective composite materials for medical and healthcare purposes.

## 2. Fields of Application for NPs/Polymer Composites

Among the biomedical applications of NPs/polymer composites developed during the last five years, many different directions can be seen (Table 1). Figure 1b shows that the most popular direction (31%) is the development of materials for wound treatment. Here, new antibacterial composites for wound dressings are under active development [3,4,5,6,7,8,9,10,11,12,13,14,15,16], along with materials for wound healing [17,18,19,20,21,22,23,24,25,26,27]. Materials for wound infection therapy [28], especially those for fighting drug-resistant bacterial infections [29], are also described. The treatment of chronic wounds that have prolonged healing time is one of the most important tasks in this direction, which challenges researchers [30,31]. Active wound dressing systems [32] and multifunctional materials for skin wound repair [33] are developed as well. Chen and co-authors [34], for example, prepared composite nanofiber membranes for medical wound dressing that combine antibacterial, anti-inflammatory, and antioxidant actions. Miralaei etc. [35] developed composite biofilms for rapid wound dressing, which among others are able to control bleeding. Composites for wound healing and blood clotting were also reported by Alizadeh and co-authors [36].

After wound treatment, the next most popular application for NPs/polymer composites is medical devices (12.9%). Antibacterial materials described here can be used in either a wide range of different devices and disposable medical equipment [50,54,71,74,98], or have specific applications, for example, for orthotic [43,79] and orthopedic devices [68], for prosthetics [43,53,110] and implants [108], for medical gloves and multipurpose rubber sheets [86], and for catheters [31,51,61].

The third place in the analyzed fields belongs to the antibacterial NPs/polymer composites for bone and tissue engineering (9.5%). Among the materials developed in this direction, there are agents for tissue engineering [80,95], and more specifically, for bone tissue engineering [64,69,102], bone regeneration [42,59,99], and bone reconstruction [109]. For example, Benedini et al. reported on alginate/nanohydroxyapatite composites that can be potentially used as antibacterial bone filler [107], Table 1. Composition and preparation methods for the antibacterial NPs/polymer composites are under review.

The materials for skin tissue engineering are also described in the literature [13], with one of the best examples being a conducting polymer/silver NPs composite as a potential xenograft for burn treatment [32].

Antibacterial coatings made of NPs/polymer composites occupy the fourth popular place (9.9%) in the field under view (Figure 1b). In this direction, mostly antibacterial coatings for non-specified biomedical purposes are developed [45,46,48,87]. Multifunctional antibacterial coatings with self-healing properties can be found [37,66], with additional specific coatings mentioned. For instance, Chacko with co-authors [112] prepared a composite system with bacterial-resistant and magnetoelectric properties that can be used in electric and magnetic field-tuned coatings, which are ideal for implantable medical devices and smart textiles for healthcare uses.

Composites of NPs and polymers developed in the last five years also include materials for dental applications (6%). Dental composite resins [55,85], including compositions with self-healing capabilities [103,105], and systems with therapeutic [56], defect repairing [81] and restorative filling [104] capabilities were reported to be developed in this direction.

NPs/polymer composites with antibacterial properties are also used for different wearables (3.4%). Here, materials for wearable electronics [52,58], flexible wearable strain sensors [39], and textile sensors for wearables and smart clothes [65] can be mentioned.

Antibacterial composites for drug delivery are another direction for the development of NPs/polymer systems, which occupy some 2.6% of publications in the field; these include research on materials for drug delivery [40,101] and scaffolds for drug sustained-release through direct coating or blending [60].

About 22.4% of the authors did not specify the applications for their developed materials, only indicating that they were aiming at preparing materials for biomedical [5,44,47,76,77,83,88,89,91,92,93,94,97,113,114,115] and healthcare [70,73,84] uses. Some studies were aimed at the preparation of materials that can act as advanced antibacterial agents [62,63], to reduce the future risks of conventional antibiotic misuse [90] and to manage drug-resistant bacteria [31] and biomaterial-centered infection [72].

The other articles reviewed (5.2%) were found to be dedicated to very specific applications. Among them, there is an antibacterial material for use as a hemoperfusion adsorbent for efficient bilirubin removal [96]. A material for the treatment of visceral leishmaniasis is also described [116]. Datta et al. [57] developed a material that has a strong effect against Shigella. Bashal with co-authors [95] prepared a composite that has potential for pharmaceutical applications. MRI imaging with a cytocompatible and antibacterial agent was proposed by Kafali et al. [100]. Banerjee with co-authors reported on films that can be used as top-sheets in feminine sanitary hygiene napkins [82].

Thus, it can be concluded that the NPs/polymer composites developed over the last 5 years have a wide variety of different biomedical applications, starting with wound dressing and ending with xenografts and MRI.

## 3. Nanoparticles for Composites

The NPs incorporated into the polymer matrix provide not only antibacterial properties but can also enhance the mechanical performance of the polymer matrix [86], improve the pore size distribution of the matrix [96], and affect the electric conductivity [33,84]. Moreover, they can provide additional cross-linking of the polymer components [86], adjust semiconductors properties [84], bring a self-healing effect [41], enhance the composite functional properties [81], and so on. Thus, the role of NP filler in the composites under consideration is quite multifunctional.

**Figure 2:**
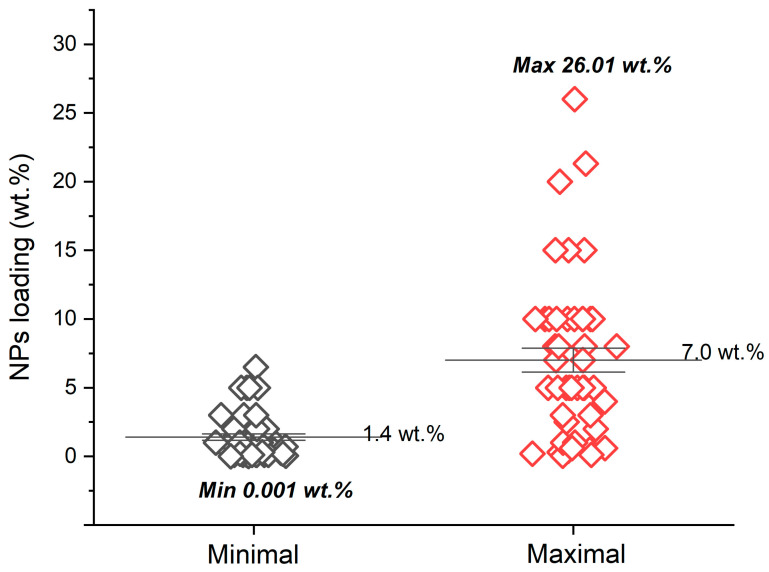
Minimum and maximum concentrations of NPs loaded into NPs/polymer composites, as reported in 72 reviewed articles.

The dispersion of particles at the nanoscale level is known to result in a very high interfacial area for interaction of polymer chains with NPs. This can lead to enhancement of certain properties even at a low loading of the filler [87]. The amount of filler added to composites differs among studies. Typically, researchers prepared composites with different loadings of NPs and compared the properties of the resulting materials. The loading ranges (minimal and maximal content of NPs) are graphically presented in Figure 2. It is seen that the average minimal loading was 1.4 wt.%, and maximal was 7.0 wt.%. The overall minimal concentration found was 0.001 wt.% of NPs [110], and the overall maximal loading was 26.01 wt.%, reported in the work by Luo [24].

In the following sections, we discuss and summarize the types of fillers and their origination and composition mentioned in the literature.

### 3.1. Type and Composition of Filler

**Figure 3:**
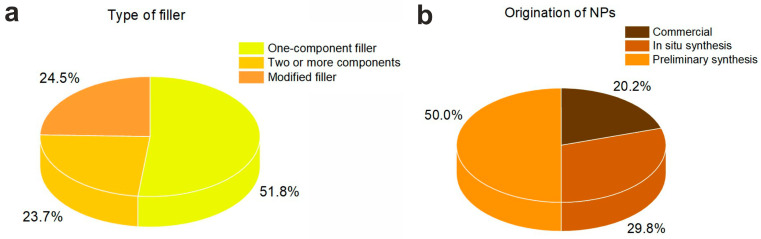
Pie diagrams on the type of filler (**a**) and origination of NPs (**b**).

This review focuses on composites containing at least one kind of inorganic NP as the filler. In this respect, the composites covered here can be classified as containing one-component filler or complex filler (more than one component). Complex fillers, in turn, can be divided into two groups. The first group contains fillers represented by two or more different materials (at least one of them being in a form of NPs). The second group is fillers that contain NPs modified with different modifiers. The resulting percentage of each type can be seen in Figure 3a. It is seen that, at present, the one-component fillers are preferable in such antibacterial NPs/polymer composites (51.8%). Multicomponent and modified fillers were reported by almost the same number of surveyed reports (23.7 and 24.5%, respectively).

**Figure 4:**
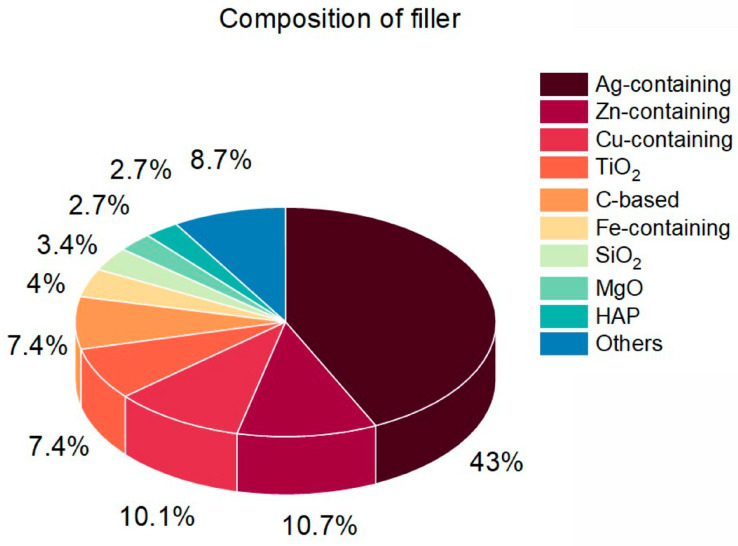
Pie diagram indicating the type composition of the filler.

As for the composition of fillers, silver-containing particles are seen in Figure 4 to occupy the leading position (43%) in the field. Concerning this, in 61 out of 64 articles, composites with Ag NPs are prepared (see, for example, [37,39]). The other articles on Ag-containing fillers are devoted to Ag_2_O [53], AgBr [31], and Ag_2_[Fe(CN)_5_NO] [26] NPs. The second place in Figure 4 is occupied by Zn-containing fillers (10.7%). Here, 14 out of 16 published articles deal with ZnO NPs fillers (for example, [79,81]). About 10.1.% of works published reports on Cu-containing NPs, with CuO NPs occupying 8 out of 15 positions (for example, [87]). The other articles devoted to Cu-containing NPs mentioned Cu [87,88,92], CuS [90], Cu_2_O [89], and CuFe_2_O_4_ [89] NPs. The same percentage (7.4%) belongs to the works that consider TiO_2_ (for example, [9,16]) and carbon-based fillers. The latter type of fillers is mostly represented with graphene oxide (GO, see, for example, [65,67]), and then there are graphene [71], C_3_N_4_ [66], carbon nanotubes [63], and carbon quantum dots [98]. In the case of Fe-containing fillers (4%), about half of the papers found consider Fe_3_O_4_ magnetic NPs (for example, [28,100]), with the other half devoted to LiFe_5_O_8_ [112], CuFe_2_O_4_ [89], and Ag_2_[Fe(CN)_5_NO] [26]. Silica [35,105], MgO [106], and hydroxyapatite (HAP) [59,97] were reported as fillers with roughly the same frequency (3.4%, 2.7%, and 2.7% of the pie, respectively). Finally, the other fillers, including noble metals (Au NPs [33], Pt NPs [3], Pd NPs [3]); CoO [111]; Al_2_O_3_ [110]; NiO [116]; CaO [113]; Te [115]; CeO_2_ [99]; glass [108,109] NPs; etc., are seen in Figure 4 to occupy the remaining 8.7%.

So, it is seen that over the last five years, the research on antibacterial NPs/polymer composites tended to focus on one-component fillers, predominantly containing silver. However, more complex and complicated fillers based on other metals, metal oxides, etc., were also tested and applied for different biomedical purposes.

### 3.2. Origination of Nanoparticles

To obtain NPs/polymer composites for medical applications, either commercial or home-made NPs can be used. The latter NPs could be both synthesized in situ (during composite formation or in the presence of the pre-prepared polymer matrix) or preliminarily prepared before fabricating composite. Some examples for all these cases can be found below.

Commercial NPs were reported in 20.2% of reviewed articles (Figure 3b). Such NPs could be used either as-supplied (for example, [36,42,111]) or modified (for example, [96,102,103,105]), or after additional treatment (for example, [97]). Modification is made with different purposes. For example, Ahangaran and co-authors [103] modified SiO_2_ NPs with 3-methacryloxypropyl-trimethoxysilane in order to enhance their dispersion inside the acrylic matrix and to improve their adhesion in it. This permitted the derivation of a new self-healing dental composite. In the work by Du et al. [96], TiO_2_ NPs were modified with vinyl-triethoxysilane for the sake of improving its hydrophobicity, along with formation of double-bond composite with styrene, which was used as a novel antibacterial bilirubin adsorbent with high bilirubin clearance capacity. Modification of MgO NPs with FeCl_3_ and tannic acid was reported to lead to the formation of a composite with both good antibacterial activity and excellent photothermal effect [102]. Additional treatment can be made to further decrease particle size, as was reported, for example, by Babers et al. [97], who converted commercial micro-sized particles of TiO_2_ into nanosized ones by means of grinding.

**Figure 5:**
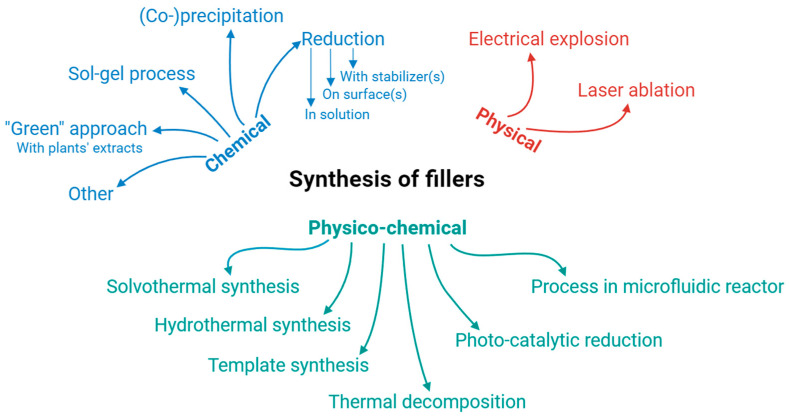
Methods used by the authors of reviewed publications to prepare their inorganic NPs (as composite fillers).

However, the majority (79.8%) of researchers working on developing new NPs/polymer composites preferred to prepare NPs instead of using commercial products. The methods used to prepare NPs for NPs/polymer composites are schematically presented in Figure 5. It is clearly seen that a wide spectrum of chemical, physical, and physico-chemical approaches known for inorganic NPs were applied by the authors of the articles.

Synthesis of NPs prior to preparing the composite was a choice of 50.0% of the authors. Both chemical and physical preparation methods were described in the articles under review. The most popular preparation techniques were wet-chemistry approaches [6,15,22,35,44,59,63,70,76,79,100,105,114], including precipitation [72] and co-precipitation [101] techniques, chemical reduction [5,10,13,29,41,43,50,55,57,67,68], and chemical synthesis, where a polymer acted as NPs’ stabilizer [38,54,61,62,112]. Moreover, sol–gel synthesis [77,84,85,108,109], solvothermal [90], and hydrothermal [92,99] approaches were described. Green synthesis [9,12,17,59], biosynthesis [4,32], and template-synthesis [104] can also be found in the reviewed articles. Among physical and combined physical–chemical methods used to prepare NPs for composites, thermal decomposition [60], electrical explosion [89], and laser ablation in liquids [7,74,87,110] can be mentioned.

In situ preparation of NPs during composite synthesis or directly in the presence of polymer matrix was also a popular approach; about 29.8% of articles considered NPs prepared in situ. One-pot preparation of composites seems to be quite simple and controllable in terms of NPs size and distribution inside the polymer matrix, along with their better fixation. For example, in the work of Jia and co-authors [28], Ag NPs were obtained on the surface of FCE hybrid (Fe3O4NPs/carboxymethyl cellulose-ε-polylysine) via chemical reduction. The abundant functional groups available in the polymer matrix resulted in good anchoring of Ag NPs inside the prepared FCE hybrids.

The one-step method reported by Chen with co-authors for in situ reduction of Ag+ directly in the spinning solution was found to significantly simplify the preparation process of Ag NPs/PVA/PAA composites [34]. Importantly, it also decreased the amounts of raw material waste, thus making the process “greener”. As for the product, such an approach helps avoid the aggregation of Ag NPs. Luo et al. co-polymerized cheap and industrially produced imidazole-2-carbaldehyde and melamine, obtaining porous organic polymer that had a very high content of N (almost 40 at.%) [24]. This allowed them to effectively anchor Ag on the porous skeleton with quite high loading (26 wt.%) without aggregation. Also, such an approach helped greatly to reduce the toxicity of the Ag NPs.

Laser ablation in situ was also used to prepare NPs/polymer composites. For example, laser ablation of a copper target in a polyvinyl alcohol (PVA)/polyvinyl pyrrolidone (PVP) blend was carried out to form a composite with enhanced antibacterial activity for wound healing [20]. Haladu et al. [40] applied laser ablation in situ to form silver quantum dots (Ag QDs) in polyaspartate, with the composite resulting in well-dispersed Ag QDs in the polymer matrix, which was shown to have potential as an antibacterial agent for drug delivery.

Thus, the majority of authors working on new antibacterial composites of NPs in polymer preferred to use preliminarily prepared NPs. In situ preparation of NPs was also reported, while commercial NPs were considered within about a quarter of reviewed works.

## 4. Polymers for Composites

Polymers are unique materials with specific properties which are particularly attractive for biomedical applications. They are non-toxic, sustainable, renewable, very adaptable, tunable, and well compatible with biosystems, having low risk of rejection and adverse reactions [84,88,99]. They can be produced with tuning of desired specific properties, such as flexibility, conductivity, mechanical strength, thermal behavior, degradation rates, permeability, and processability [84,87]. They can also be filled with different fillers and, which is of most importance, they can be prepared in different forms, starting with protective coatings and films (for example, [49,86]), and ending with nanofibers [34], and patient-specific implants [108].

In the following sections, the types of polymer matrices together with their composition and preparation methods described by the researchers working on novel NPs/polymer composites are considered.

### 4.1. Polymer Matrix Composition

**Figure 6:**
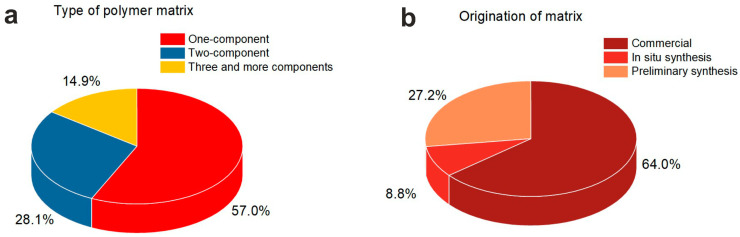
Pie diagrams showing the type of polymer matrix (**a**) and its origin (**b**).

The list of polymers used for obtaining NPs/polymer composites for medical purposes is quite long. All of the diversity in the matrix compositions can be divided into two groups, namely, one-component polymer matrices, and complex ones. The group of complex matrices combines two-component matrices and matrices of three or more components (Figure 6a). It can be noticed that one-component matrices (for example, [17,82]) are more common (57.0%). Two-component matrix compositions (for example, [7,96]) were used in 28.1% of works, while only 14.9% of the authors working on new NPs/polymer compositions used polymer matrices consisting of three or more components (for example, [16,51]).

The majority of polymers used in antibacterial composites for biomedical applications are listed in Figure 6 and Table 2 and Table 3, whereas the basic properties of the polymers are presented in Table 2. Table 3 provides information on the polymers’ features that are of particular importance for biomedical applications.

**Figure 7:**
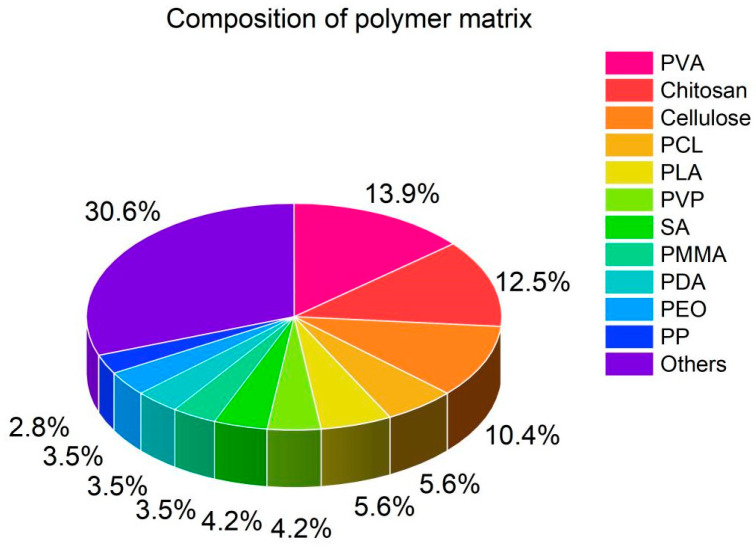
Pie diagram showing the polymer matrix composition.

There are leaders among polymers that were most commonly mentioned in the literature (Figure 7). Polyvinyl alcohol (PVA) was used for the formation of polymer matrix in 13.9% of reviewed articles (for example, [9,20]). PVA is a semi-crystalline, non-toxic, and low-cost polymer with excellent flexibility, transparency, and hydrophilicity. It is also soluble in hot water, which makes it even more attractive for use. Chitosan was used by 12.5% of the authors (for example, [49,88]). Chitosan is a vital polysaccharide with unique biological activity. It contains different reactive groups (primary amine groups, primary hydroxyl groups, secondary hydroxyl groups), which are the source of the chitosan specific bioactivity. It is also non-toxic, biocompatible, and possesses antibacterial properties. The third place (with the score of 10.4%, see Figure 7) belongs to cellulose (for example, [52,84]). It is a biodegradable, sustainable, renewable, and biocompatible polymer. An abundance of surface hydroxyl groups results in extensive hydrophilic properties of cellulose. Its applicability for biomedical purposes cannot be overestimated. Polycaprolactone (PCL) was used by 5.6% of the authors (for example, [82,99]). PCL is a synthetic polymer which possesses such beneficial properties as excellent biocompatibility, good mechanical properties, flexibility, thermal stability, and low melting point (60 °C). Also, it has good blend ability with other polymers, and it can be easy functionalized. Polylactic acid (PLA) occupies 5.6% of the pie in Figure 7 as well. It shows favorable biocompatibility and biodegradability, along with shape-memory properties [70,102].

Polyvinylpyrrolidone (PVP, with its 4.2% fraction in Figure 7) is a low-toxic, biodegradable amorphous polymer. Its films are easy-forming and can act as a protective agent for different surfaces [20]. Sodium alginate (SA, mentioned in 4.2% of works) is a cheap, hydrophilic, biocompatible, and biodegradable polymer. Also, it is well tolerated by the immune system of humans [9]. Polymethyl methacrylate (PMMA, mentioned in 3.5% of publications) exhibits good biocompatibility, physical and chemical stability, affordability, and malleability, which is extremely desired for, for instance, modeling of innovative orthopedic prostheses [79,89]. Polydopamine (PDA, also used in 3.5% of studies) is a durable and biocompatible coating material that can be used for functionalization and modification [49,52]. Polyethylene oxide (PEO, used in 3.5% of articles) is quite often used as a matrix-forming agent (for example, [22,29]). Polypropylene (PP) is a thermoplastic with high distortion temperature and low density. It is transparent, flame resistant, corrosion resistant, stable and easily reprocessed. It was used by the 2.8% of the authors (for example, [47,92]).

There are also other polymers (30.6%) used for the matrix formation, such as polyaniline [90], polyurethane [51], gelatin [13], polyethylene [76], polyvinylidene fluoride [69], polystyrene [96], fibroin [3], and so on.

Thus, it is seen that in recent years the studies devoted to novel NPs/polymer composites dealt preferably with one-component matrices, with the most popular polymers being PVA, chitosan, and cellulose. However, much more complex systems and rarer polymers were also applied.

### 4.2. Origination of the Polymer Matrix

**Figure 8:**
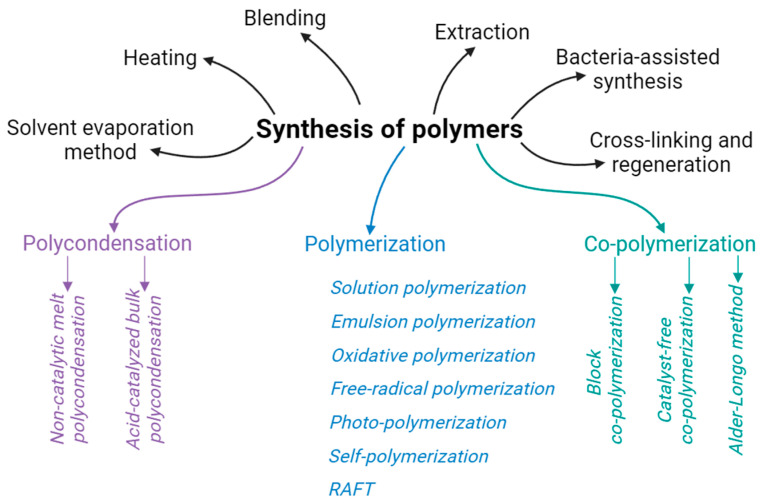
Methods used by the authors of the reviewed publications to prepare their polymers (as composite matrix).

The polymer matrices used for composites were prepared either from commercial polymers or from homemade materials. In the latter case, polymerization (or other ways of preparing polymer matrix) was carried out either in situ or prior to the composite formation. Figure 6b shows that commercial polymers were used the most frequently (64.0%), while preliminary preparation and in situ polymer synthesis were applied by 27.2% and 8.8% of the authors, respectively. The methods used for polymer preparation are exhibited in Figure 8, where essentially all methods known for polymer preparation can be seen.

To obtain a polymer matrix for the composite from commercial polymers, the solution mixing method [10,60,62,94], reactive melt mixing [47], latex processing technique [86], and others were employed. Among the methods used to obtain polymers “in-house” were polymerization and co-polymerization [24,26], solution [31] and emulsion polymerization [41,48], poly-condensation [40,71,109], and reversible addition-fragmentation chain transfer (RAFT) polymerization [27,56,73]. As for preparation of the polymer matrix during composite formation, in situ polymerization was typically applied [43,90,96,105,107]. Also, NPs could be obtained in situ on the surface of a pre-prepared polymer matrix [28,33,46,50,58].

**Figure 9:**
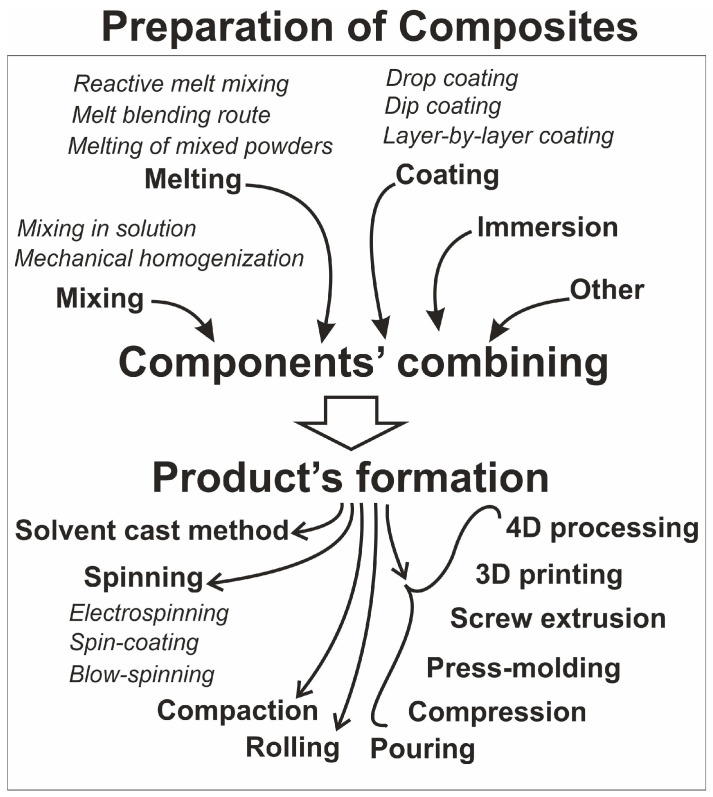
Methods used by authors of reviewed publications to prepare their composites (filler in polymer matrix).

Figure 9 presents the methods that were used for composite preparation. The first stage of the process was typically combining the components, i.e., polymer(s) and filler(s), after which the product’s formation was performed.

To prepare a resulting product in a form of composite film, the solution or solvent casting approach was used [4,6,9,72,83,89,99,111]. Wang et al. [49] used simple immersion of the substrate (catheter) in a solution with reagents to prepare very uniform composite antibacterial coatings. To obtain composite scaffolds or mats, electrospinning was often applied [3,8,17,22,30,34,80,82]. For composite scaffold formation, the selective laser sintering process could also be used [68]. Babers et al. [97] produced composite sheets via the compaction technique. Su and co-authors prepared composite fibers by the solution blow-spinning process [70]. Also, 3D printing [98,108] and 4D processing [102] were reported for production NPs/polymer composites for biomedical applications.

Thus, commercial polymers were most frequently used to obtain antibacterial NPs/polymer composites. At the same time, preliminarily prepared and in situ synthesized polymers were also applied.

## 5. Antibacterial Activity of NPs/Polymer Composites

### 5.1. Comparative Antibacterial Activity of the Composites

An attempt to compare the antibacterial activity and cytotoxicity (in terms of cell viability) of the materials described in the reviewed articles was also made. For this, relative values of the mentioned parameters were calculated as follows:

$$Relative\;P,\%=P_{n}/P_{0}\cdot 100-100,$$

where P is the parameter (antibacterial activity or cell viability), P_n_ is a value for the composite material, and P_0_ is a value for the polymeric material without filler.

**Figure 10:**
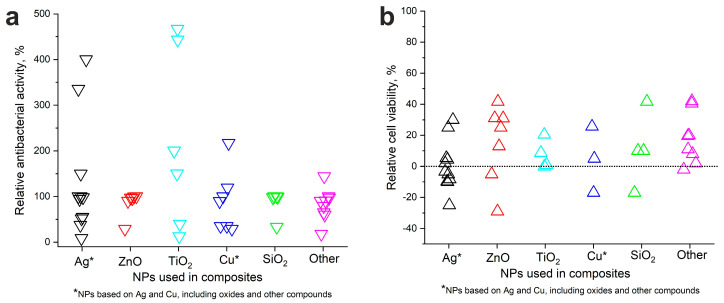
Relative antibacterial activity (**a**) and cell viability (**b**) of composites as a function of load for different fillers (NPs).

Values of P_n_ and P_0_ parameters were obtained from the articles (note that not all the authors provided such data). The results of calculating relative P parameters related to different filler NPs are presented in Figure 10.

In general, it can be noted that the highest relative antibacterial activity was shown by composites loaded with TiO_2_-, Ag-, and Cu-based nanosized fillers (see Figure 10a). Furthermore, Figure 10b shows that these three types of fillers could also exhibit negative relative cell viability. This implies that loading fillers such as Cu, Ag, and TiO_2_ can lead to lower cell viability (and higher cytotoxicity). Thus, researchers working on developing systems that incorporate highly antibacterial active fillers must find an optimum between antibacterial activity and cytotoxicity.

### 5.2. Antibacterial Mechanisms of the Composites

**Figure 11:**
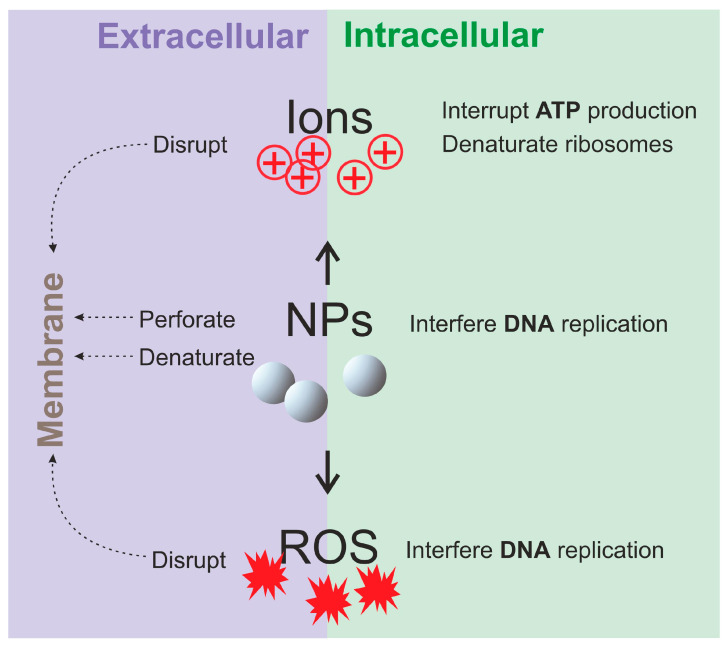
Mechanisms of antibacterial action/activity known for inorganic NPs.

The existing mechanisms known for NPs that demonstrate antibacterial activity are schematically presented in Figure 11. Such antibacterial activity was found to be based on the action of NPs themselves, and/or on metal ions they release, and/or on the action of reactive oxygen species (ROS) generated in their presence. All of these agents can potentially influence the microorganism’s cells, both extracellularly and intracellularly. The concrete effects of NPs, as well as of ions and ROS released or produced by them are listed in the scheme below (Figure 11).

Since Ag-based filler is the most popular for reviewed NPs/polymer composites (see Section 3.1.), antibacterial action via Ag NPs penetration of the cell membranes and Ag ions release are met very often in the literature (for example, [25,50]). But intensive release of Ag ions can be harmful for human cells. Immobilization of Ag NPs onto different substrates can help avoid the side effects of silver. For this, Jatoi with co-authors [62] immobilized Ag NPs onto TiO_2_ NPs and then prepared their composite with cellulose acetate nanofiber. Thus, silver ions and NPs themselves acts as antibacterial agents.

Then, the oxidative stress of bacteria is described by Sun et al. [45] as a possible main mechanism of the bactericidal activity of chitosan composite with Ag NPs. In the work by Du et al. [96], bacteria were found to adsorb onto the composite, and TiO_2_ under UV-light excitation produced reactive oxygen species (ROS) that killed the bacteria. Sebak et al. [94] suggested that TiO_2_ NPs interact with the cell membranes and penetrate inside the bacteria, releasing ROS.

Along with TiO_2_ particles and Ag NPs and ions, other NPs and ions were also reported to provide antibacterial properties to composites. Glazkova with co-authors [89] demonstrated three possible antibacterial mechanisms of CuFe_2_O_4_/Cu_2_O/CuO/PMMA composite, namely, NPs interaction with bacteria, copper ion release, and ROS generation. Jardon-Maximino et al. [91] pointed out that the ability of composite to release Cu ions was in agreement with its antibacterial properties, so functionalized Cu NPs were the source of the ions.

Liu et al. [83] showed that ZnO modified with citric acid monohydrate (ZnO-CA) could only exhibit antibacterial effect when it was in direct contact with bacteria. Incorporation of such NPs into PCL matrix, which gradually degraded exposing more ZnO-CA, was shown to result in a long-term antibacterial effect. Abdelaziz et al. [59] revealed that the inhibiting activity of Ag NPs-loaded nanofiber increased with time and maximal effect was observed after 32 days. Nanofiber of polylactic acid/cellulose acetate and poly(caprolactone) polymers degraded and higher concentrations of Ag NPs became available. Similar long-term antibacterial activity was described by Guo with co-authors [60] for scaffolds with sustained release of Ag^+^. Haladu et al. [40] described synergistic interaction between polyaspartate (PASP) and Ag QDs. PASP biocompatibility was due to electrostatic adsorption of amino groups on the membranes of bacteria. Then, the polymer provides a long-term release of Ag ions for prolonged antibacterial action.

Many examples of synergistic effects were found in the reviewed articles. Bagheri et al. [18] revealed that the presence of both ZnO and Ag NPs in the composite scaffold showed a stronger synergistic antibacterial effect compared to the control antibiotic and bare polymeric disks. Hezma and co-authors [77] pointed at the fact that the antibacterial activity increase was a result of synergy between co-polymer CS-PVA and ZnO NPs via reactive oxygen species (ROS) formation and Zn^2+^ release, respectively. Astashev et al. [110] described a synergistic effect, in which borosiloxane matrix protects NPs of different influences (physical and chemical), while aluminum oxide NPs accelerated the formation of ROS. Such a synergistic effect allowed a reduction in the amount of incorporated NPs without a decrease in the antibacterial activity. In the work of Jia and co-authors [28], cellulose-based composite with Fe_3_O_4_ and Ag NPs showed the best antibacterial results with the assistance of H_2_O_2_, indicating the synergistic strategy. Guo et al. [21] hypothesized the following factors for outstanding synergistic antibacterial capability of polyCu-MOF@AgNPs composite: intracellular ROS production, Ag ion release under acidic conditions, entrance of Ag NPs and metal ions into the cell, morphological change in bacterial membrane, and damage of DNA or proteases.

Along with synergistic effect, a combined action of the composite’s components can be the basis for its antibacterial activity. Joy et al. [99] suggested that the antibacterial effectiveness of the PCL-GO-CeO_2_ composite is based on the combined action of the filler’s components. Thus, CeO_2_ generates ROS, and GO physically destroys the bacterial cell membranes by its sharp edges. In the work by Shah et al. [90], the combined effect of CuS and PANI was described as the factor improving the antibacterial activity via ROS generation.

The structure of the composite can also be an important factor for antibacterial activity. Su and co-authors [70] pointed out that the pore structure of the composite fibers plays quite a significant role in antibacterial activity. The release of Zn^2+^ and Ag^+^ is facilitated by high porosity and specific surface area, and causes generation of a large number of ROS, which induce oxidative stress in bacteria. Also, in the work by Wang et al. [71], the structural effect on antibacterial activity appeared in a form of maximizing the loading of Ag NPs via increasing the accessible surface area of the substrate material, and its highly porous structures. They used in situ reduction of Ag^+^ by tannic acid (TA) and revealed the TA concentration-dependent antibacterial activity (as more TA could reduce more silver ions). Another structure-based antibacterial effect was described by Balan et al. [95]. The poly-ortho-toluidine-TiO_2_/PCL scaffolds’ porosity and hydrophobicity improved the bacteria interactions with substrate, leading to antibacterial activity enhancement.

Other properties can also affect the antibacterial behavior of the composite. For example, in the work of Chacko and co-authors [112], the PVDF and micro-crystalline cellulose-based composite with LiFe_5_O_8_ NPs showed a correlation between antibacterial properties and electro-active nature. So, the electrical activity of the material can also play some role in hindering bacterial growth.

Thus, different mechanisms of antibacterial activity can be found in the reviewed works. The described mechanisms are mostly complex and may include both effects of NPs and polymer matrix. New synthetic approaches for preparation composites with structure-dependent effects, combined antibacterial action of components, and synergistic effect result in excellent bactericidal and inhibition effects of NPs/polymer composites for biomedical applications.

## 6. Conclusions

Thus, it can be concluded that antibacterial NPs/polymer composites developed over the last 5 years have a wide variety of different biomedical applications, starting with wound dressing and ending with xenografts and MRI.

Within this period of time, the main trend in developing NPs/polymer composites was combining one-component filler and one-component polymer matrix. The most commonly used filler was Ag-containing NPs; the most popular matrices were based on PVA, chitosan, and cellulose. Concerning this, NPs produced “in-house” and commercial polymers were used more frequently. The average range of filler loading was found to be 1.4–7.0 wt.%, while the overall minimal and maximal concentrations used were 0.001 wt.% and 26.01 wt.%.

Based on analysis of the reviewed articles, the growing trend was found on trying new, cheaper, and more-readily available fillers, which in the future would substitute for Ag-containing NPs. Also, different methods of filler modification or combination/conjunction of different filler components has become popular. As for polymer matrices for such composites, the latest trend has been in creating new blends or complex polymeric compositions aiming to obtain composites with enhanced and multifunctional properties.

As for antibacterial properties, different mechanisms are described; they are mostly complex and may include effects from both the NPs and polymer matrix. New synthetic approaches for composites prepared with structure-dependent effects, combined antibacterial action of components, and synergistic effects result in excellent bactericidal and inhibition effects of NPs/polymer composites for biomedical applications.

For every application, there are specific challenges faced by the new antibacterial composites being developed. For wound treatment, for instance, such materials should exhibit not only antibacterial, but also antioxidant, blood clotting, and healing properties. For medical devices and tissue engineering, a balance should be achieved between required mechanical properties and bioactivity provided by the fillers, since the latter fillers affect the material’s strength and flexibility. Overall, researchers have been developing new antibacterial composites and need to find an optimum between antibacterial activity and cytotoxicity, as fillers with the highest antibacterial action are quite often highly cytotoxic. Here, different approaches for lowering toxicity should be used, starting with capsulation or partial blockage of the toxic component and ending with substitution of widely used antibacterial fillers with new non-trivial agents.

## Figures and Tables

**Table 1:** Composition and preparation methods of the antibacterial NPs/polymer composites under review.

Reference	Polymer		Filler			Composite			Application
	Composition	Origination	Composition	Origination	Size	Composition	Synthesis	Product	
Ag-based NPs									
[37]	(1) Amine-rich aminomalononitrile (AMN); (2) Homopolymer poly(B5AMA); (3) Zwitterionic compound 2-methacryloyloxyethyl phosphorylcholine (MPC); (4) statistical co-polymer poly(MPC-st-B5AMA)	All the components were separately synthesized via polymerization	AgNPs	Synthesized in situ via chemical reduction	-	AMN/B5/MPC/Ag	Components were applied to the surface one by one	Self-healing coating on glass substrates	Coating
[38]	Polyvinyl alcohol (PVA); sodium alginate (SA); carboxymethyl chitosan (CMCS)	Commercial	Ag NPs	Synthesized with SA as reductant and stabilizer	12 nm	PVA/SA/CMCS/ SA- AgNPs	Mixing in solution, immersion into CaCl_2_ solution for cross-linking	Gel	Wound dressing
[34]	Polyvinyl alcohol/polyacrylic acid (PVA/PAA)	Commercial	AgNPs	Synthesized in situ via chemical reduction	20–160 nm	PVA/ PAA/Cur/AgNPs	Mixing in solution, electrospinning	Nanofibers	Wound medical dressing
[39]	Highly elastic natural rubber (NR); polydopamine (PDA)	Self-polymerization of dopamine in the NR (commercial) emulsion	AgNPs	Synthesized in situ via chemical reduction by PDA	between 80 and 120 nm	PDA/NR/AgNPs	Mixing in solution	Film	Flexible wearable strain sensors
[7]	Carboxymethyl cellulose (CMC); polyvinyl alcohol (PVA)	Commercial	AgNPs	Synthesized via pulsed laser deposition in situ in polymer solution	-	PVA- CMC/ AgNPs	Pulsed laser ablation pf Ag target in CMC-PVA blend	Film	Wound dressing
[19]	Polyvinyl alcohol (PVA); Polyvinyl pyrrolidone (PVP)	Commercial	AgNPs	Synthesized via laser ablation in situ	45.3 to 81.3 nm	PVA/ PVP/ AgNPs	Pulsed laser ablation pf Ag target in PVA/PVP blend	Film	Wound healing/ dressing
[32]	Polypyrrole (PPy); polyaniline (PANI)	Polymerization	AgNPs	Biosynthesis with citronella oil	35–50 nm max at 45.64 nm	TS/PPy/ AgNPs TS/PANI/AgNPs	Nile tilapia skin (TS) was covered step-by-step with polymers and NPs	Coating	Xenograft for burn treatment; active wound dressing system
[21]	Copper-based polymer–MOF (polyCu-MOF)	Synthesized using polyether containing specific units as building block, 4,4′-bipyridine as co-ligand, and copper ions as metal-coordinated centers	AgNPs	Synthesized in situ via chemical reduction	-	polyCu-MOF@ AgNPs	Chemical reduction of Ag ions on the polyCu-MOF matrix	Scaffold	Wound healing
[40]	Polyaspartate (PASP)	Polycondensation of L-aspartic acid	silver quantum dots (Ag QDs)	Synthesized via laser ablation in situ	5 nm	PASP/ AgQDs	Pulsed laser ablation pf Ag target in PASP solution	Powder	Drug delivery
[10]	Polyvinyl alcohol (PVA)	Commercial	AgNPs	Synthesized via chemical reduction	~116 to 208 nm	PVA/ Ag NPs	Mixing in solution	Film	Wound dressing
[12]	Inner layer: PVA, chitosan, gelatin; Outer layer: polyacrylonitrile (PAN)	Commercial	AgNPs	Green synthesis with Capsella bursa-pastoris extract	72.57 nm	Inner layer: PVA, chitosan gelatin/AgNP outer layer: polyacrylonitrile (PAN)/AgNPs	Mixing in solution, electrospinning	Two-layer nanofiber mats	Wound dressing
[22]	Polycaprolactone (PCL); chitosan (CH); poly(ethylene oxide) (PEO)	Commercial	AgNPs	Synthesized via photocatalyzed reduction	27 ± 4.3 nm	PCL/CH/PEO/AgNPs	Layer-by-layer electrospinning PCL, then CH/PEO; after that drop-coated with AgNPs colloid	Composite membrane	Wound treatment; regenerative medicine
[14]	Bacterial cellulose (BC)	Obtained using Komagataeibacter intermedius (MBS-88)	AgNPs	Synthesized in situ	5–25 nm 17.5 nm	BC/AgNPs	BC membrane poured with silver undergone hydrothermal reduction	Mats	Wound dressing
[41]	Random co-polymers of methyl methacrylate and butyl acrylate (MMA-co-BA)	Emulsion polymerization	AgNPs	Synthesized via eco-friendly reduction	5–30 nm	MMA-co-BA/AgNPs	Mixing in solution	Multifunctional film with photo-activated healing	Coating
[23]	Lignin-based nonisocyanate polyurethane foam	Polymerization and blending	AgNPs	-	-	Lignin-based polyurethane foam AgNPs	Polymerization in the presence of AgNPs	Foam	Wound healing
[42]	Polylactic acid (PLLA); polydopamine (PDA); chitosan (CS)	PLLA and CS commercial; PDA polymerized in situ	AgNPs	Commercial	-	PLLA/Ag@PDA@CS/Ag	Electrospinning, dip coating, electrospinning	Fiber	Bone repair material
[24]	Imidazole-based porous organic polymer (IM-POP)	Co-polymerization via catalyst-free Schiff base chemistry	AgNPs	Synthesized in situ	-	IM-POP-Ag	-	Particles	Wound- healing
[43]	Silicon elastomer matrix Ecoflex 00-50 (Smooth-On); Polysorbate 80 (Tween 80) as a surfactant	Polymerized in situ	AgNPs	Synthesized via chemical reduction	5–50 nm	Ecoflex 00-50/AgNPs	Elastomer matrix polymerized in the presence of AgNPs	Composite matrix	Prosthetic and orthotic devices
[44]	Carboxymethyl cellulose (CMC); chitosan (CS)	Commercial	AgNPs	Synthesized from AgNO	5 to 23 nm	CS/CMC/AgNPs	Mixing in solution	Thin films	Non- specified
[45]	Chitosan (CS)	Commercial	AgNPs	Synthesized in situ	-	AgNP/CS	In situ silver reduction; plasma treatment; solvent-casting process	Film	Coating; wound dressing
[46]	P(DMAEMA-coOEGMA-OH)	Synthesized via polymerization	AgNPs	Synthesized in situ	32 ± 3 nm	P(DMAEMA-coOEGMA-OH)/AgNPs	The AgNPs were formed within the layers via the reduction by amine groups of PDMAEMA	Star nanolayers	Coating
[47]	Polypropylene (PP); polyvinyl alcohol (PVA)	Commercial	AgNPs	Synthesized in situ via reduction	-	PP/AgNO_3_/PVA	Reactive melt mixing method; MEX 3D printing	Filament	Non- specified
[48]	Popcorn-like polymeric nanoparticles (PPNPs)	Obtained by cross-linking and regeneration of polyglycidyl methacrylate (PGMA), styrene (St) and methacrylic acid (MAA)	AgNPs	Synthesized in situ	3-45 nm on A-PPNPs 25–130 nm on PPNPs	Ag@PPNPs Ag@A-PPNPs (aminated)	AgNPs obtained in situ on PPNPs via reduction	Popcorn-like particles	Coating
[49]	Polydopamine (PDA); chitosan (CS)	CS commercial; PDA polymerized in situ	AgNPs	Synthesized in situ	-	AgNPs/ PDA/CS	Substrate coated with PDA/CS, than AgNPs reduced	Coating on urinary catheter	Biomedical devices
[50]	Polyethyleneimine (PEI)	Commercial	AgNPs	Synthesized via chemical reduction	8 nm	Ag NPs/ PEI/MAO	PEI/MAO immersed into AgNPs dispersion	Coating on the Mg alloy	Medical devices
[31]	Poly(glycidyl butyl amine) (PGBA); chitosan (CS)	PGBA prepared by solution polymerization and decorated in solution by purified commercial CS	AgBrNPs	Synthesized in situ	below 20 nm	AgBr@ PGBA- C/N	AgBr chemically synthesized in the presence of matrix	Porous sheet-like structures	Non- specified
[51]	Polydopamine (PDA); polyhexamethylene guanidine (PHMG); polyurethane (PU)	PDA polymerized in situ; PHMG and PU commercial	AgNPs	Synthesized in situ	-	PU-PDA-Ag-PDA-PHMG	Step-by-step immersion of PU into solutions: polymerization of PDA, then reduction of Ag, and covering with PHMG	Composite coating on polyurethane	Reducing catheter- associated infections
[52]	Cellulose paper (CP); polydopamine (PDA); Vitrimer glass polymer (V)	CP commercial; PDA and V synthesized via polymerization	AgNPs	Synthesized in situ via reduction	-	CP@PDA@Ag-V	Growing AgNPs inside CP modified with PDA and conjunction with V	Paper-based composite	Wearable devices
[26]	Crown ether-based porous organic polymer (CEP-POP)	Polymerization via Alder–Longo method	Silver nitroprusside nanoparticles (AgNNPs) Ag_2_[Fe(CN)_5_NO]	Synthesized in situ	-	CEP-POP/AgNNPs	Obtained via “one-pot two-step” method	Nanorod-like powder	Wound healing
[27]	Polymeric vesicles from DIPEMA, GlyMA, and mPEG-CPADB	Synthesized via RAFT polymerization	AgNPs	Synthesized in situ	-	AgNP-decorated vesicles	AgNPs obtained in situ on cross-linked vesicle membranes via reduction polyvinylpyrrolidone (PVP)	Polymeric vesicles	Wound healing
[53]	Poly(lactic-co-glycolic acid) (PLGA)	Commercial	Ag_2_O	Synthesized via laser ablation in liquid	20 to 40 nm	PLGA/ Ag_2_O NPs	Obtained via low-temperature technology	Bulk composite	Prosthetics
[54]	Poly(butylene adipate terephthalate) (PBAT)	Commercial	AgNPs polyvinylpyrrolidone (PVP)	AgNPs chemically synthesized in the presence of commercial PVP	20–50 nm	PBAT/ AgNPs	Mixing in solution; solution casting method	Film	Disposable medical equipment
[29]	Silk fibroin (SF); poly(ethylene oxide) (PEO)	SF extracted from Bombyx mori cocoons; PEO commercial	DAPT-Au NPs	Reduced in the presence of 4,6-diamino-2-pyrimidinethiol (DAPT)	2.44 nm	DAPT- Au NPs/ SF/PEO	Mixing in solution	Mixed-matrix membrane	Wound dressing
[55]	Nexcomp-META BIOMED	Commercial	Ciprofloxacin-loaded AgNPs CIP-AgNPs	Synthesized via chemical reduction and loaded with CIP	Ag 33.5 nm CIP-Ag 98.32 nm	Nexcomp-META BIOMED/AgNPs Nexcomp-META BIOMED/CIP-AgNPs	NPs were incorporated into resin composite at room temperature and were homogenized manually	Disc	Dentistry
[56]	PAPMA-b-PDMAEMA (D); PAPMA-b-PPMA (P)	Reversible addition–fragmentation chain transfer (RAFT) polymerization	MNP-AgNPs	Silver was chemically reduced in the presence of melanin nanoparticles (MNP)	15.2 nm	P/D-MNP-Ag	Conjugation of functional polymers P and D with MNP-Ag under alkaline conditions	Dual-polymer functionalized melanin-AgNPs	Oral biofilm eradication systems for dentistry
[5]	Polypropylene (PP)	Commercial	Ag NPs-CS	Synthesized via chemical reduction with chitosan	5.9 nm	Ag-NPs-CS/PP	Obtained via the melt blending route by using a twin-screw extruder	Bulk composite	Non- specified
[57]	Polyoxometalate (POM)	Commercial	Tyrosine-based polymer stabilized Ag NPs (DTP-AgNPs)	DTP prepared by RAFT polymerization; AgNPs were obtained via chemical reduction in the presence of DTP	-	DTP-AgNPs/POM	Mixing in solution	Powder	Antibacterial agent against Shigella and Shigella flexneri 2a
[13]	Gelatin	Commercial	PDA-Ag NPs	PDA was polymerized, than silver was reduced on its surface	138 nm 383 ± 14 nm (DLS data)	Gelatin/ PDA- Ag NPs	Mixing in solution	Scaffold	Skin tissue engineering
[58]	Polyester	Commercial	AgNPs/Ppyr NPs	Synthesized in situ	6–25 nm	AgNPs/ Ppyr- polyester	Polypyrrole redox-polymerized and silver reduced in the presence of fabric	Nonwoven fabric	Wearable electronics and protective coats
[59]	Polylactic acid (PLA); cellulose acetate (CA); poly(caprolactone) (PCL)	Commercial	Hydroxyapatite (HANPs); AgNPs	HANPs synthesized via chemical precipitation; AgNPs obtained via green synthesis with Callistemon viminalis extract	HANPs length 25–75 nm; width 8–35 nm; AgNPs 54–79 nm	PLA/CA/PCL/ HANPs/ AgNPs	Mixing in solution, electrospinning	Nanofibers	Bone regeneration
[18]	Chitosan (CS); polyethylene oxide (PEO)	Commercial	AgNPs ZnO NPs	Commercial	-	AgNPs/ ZnO NPs/ Chitosan/PEO	Mixing in solution, electrospinning	Nanofibrous mats	Wound dressing and healing
[4]	Poly(3-hydroxyoctanoate)-co-(3-hydroxyhexanoate) (PHO)	Synthesized in situ using Pseudomonas putida KT2440 bacteria	AgNPs CuONPs	CuONPs commercial; AgNPs synthesized in situ via reduction	-	PHO- AgNPs/ CuO NPs	PHO-AgNPs obtained in situ and mixed with CuO NPs,	Film	Wound dressing
[6]	Poly(vinyl alcohol) (PVA)	Commercial	GO AgNPs	AgNPs chemically reduced in the presence of GO	AgNPs 3.1 ± 0.8 nm	PVA/GO–AgNPs	Mixing in solution, solution casting approach	Film	Wound dressing
[60]	Poly-l-lactic acide (PLLA)	Commercial	Halloysite nanotubes filled with Ag	Nano-Ag loaded into HNTs via CH_3_COOAg vacuum negative-pressure suction, injection, and thermal decomposition	50 nm × 1 μm	PLLA/HNT@ Ag NPs	Mixing in solution	Powder for SLS scaffolds	Drug sustained- release
[61]	Polydimethylsiloxane (PDMS)	Commercial	AgNPs-lecithin modified montmorillonite gNPs@LEC-Mt	Mt commercial; AgNPs synthesized via chemical reduction in the presence of LEC-Mt	8–15 nm	PDMS/ AgNPs@LEC-Mt	Polymer solution intercalation method	Film	Medical devices
[62]	Cellulose acetate (CA)	Commercial	TiO_2_ NPs AgNPs	Commercial TiO_2_ covered DOPA and AgNPs reduced on it	TiO_2_ 20–100 nm (36.12 nm) AgNPs 4–12 nm (5.9 nm)	CA/TiO_2_/AgNP	Mixing in solution, electrospinning	Nanofibers	Non- specified
[63]	Cellulose acetate (CA)	Commercial	CNT/Ag	AgNPs reduced in the presence of oxidized commercial CNTs	-	CA/CNT/Ag	Mixing in solution, electrospinning	Nanofiber mats	Non- specified
[28]	Sodium carboxymethyl cellulose (CMC); ε-polylysine hydrochloride (EPL)	Commercial	Fe_3_O_4_ Ag NPs	Synthesized in situ via chemical reduction	-	FCE/ AgNPs	NPs were step-by-step reduced on the polymer matrix	Powder	Wound infection therapy
[64]	Arabinoxylans; acrylic acid (AAc)	Arabinoxylan extracted from *P. ovata* husk; polymeric materials were synthesized via the free radical polymerization in situ	Graphene oxide (GO); hydroxyapatite (HAp); aluminum oxide (Al_2_O_3_); AgNPs	Commercial GO, HAp, and Al_2_O_3_ loaded into polymer in situ; AgNPs reduced in situ on polymer surface	-	AgNPs-Arabinoxylan/PAA/GO/ Al_2_O_3_	Synthesized via the free radical polymerization with filler and subsequent covering with AgNPs	Silver-coated scaffolds	Bone tissue engineering
[65]	Textile composition (88% nylon and 12% Spandex)	Commercial	rGO AgNPs	GO commercial; AgNPs synthesized in situ	-	Polymer fiber/rGO/AgNPs	GO placed on the textile via drop coating; silver nitrate applied there and laser processed	Fiber	Textile sensor for wearables and smart clothes
[66]	Chitosan	Commercial	Polydopamine (PDA); carbon nitride C_3_N_4_; Ag NPs	PDA polymerized in the presence commercial C_3_N_4_; AgNPs reduced on them after that	-	C_3_N_4_- PDA-Ag@CS	Mixing in solution	Film	Wound healing
[67]	Poly(ε-caprolactone) PCL	Commercial	GO-AgNPs	GO produced by modified Hummer’s method; AgNPs reduced in the presence of GO	5 ± 0.5 nm	PCL-GO-Ag	Mixing in solution, electrospinning	Nanofibrous mat	Non- specified
[68]	Polyglycolic acid (PGA); poly-L-lactic acid (PLLA)	Commercial	Mesoporous bioactive glass (MBG); AgNPs	MBG obtained via sol-gel process, and functionalized with polydopamine; AgNPs reduced on pMBG surface	AgNPs 5 nm	PLLA/ PGA/Ag@pMBG	Mixing in solution; selective laser sintering to prepare scaffold	Scaffolds	Treatment of infected bone defects
[69]	Polyvinylidene fluoride (PVDF)	Commercial	Ag-pBT NPs	Barium titanate (BaTiO_3_) powder modified with dopamine and AgNPs reduced on its surface	AgNPs 10 to 45 nm 24.5 nm	Ag-pBT NPs/ PVDF	3D scaffolds built layer-by-layer using self-developed SLS system	Scaffolds	Orthopedics
[70]	Polylactic acid (PLA)	PLA made of commercial PLLA and PLDA	ZnO–Ag NPs	Simultaneous chemical synthesis	ZnO rods of 20 nm × 30–80 nm Ag NPs of 5–15 nm	PLA/ZnO-Ag NPs	Solution blow spinning process	Porous fibers	Personal protection and medical care
[71]	PolyAspAm(EDA/EA)/TA: polyaspartamide (PolyAspAm); thylenediamine (EDA); thanolamine (EA); tannic acid (TA)	Prepared by acid-catalyzed bulk polycondensation	AgNPs; 3D graphene	GO commercial; AgNPs synthesized in situ	less than 50 nm	PolyAspAm(EA/EDA)/TA-GO-AgNP	Mixing in solution; AgNPs obtained in situ via reduction	Nanocomposite aerogel	Medical devices
[25]	Bacterial cellulose (BC)	Prepared using G. xylinus	Graphene (GO); AgNPs; polydopamine (pDA)	GO produced by Hummer’s method; AgNPs reduced in situ	AgNPs less than 50 nm	Ag-pDA (rGO)/BC	BC (GO) formed by blending and naturally combining; then immersed in DA and soaked in AgNO_3_	Film	Wound healing
[72]	Triblock co-polymer PEG–PHBV–PEG (PPP)	Copolymerization of poly(ethylene glycol), b-poly(3-hydroxybutyrate-co-3-hydroxyvalerate) and poly(ethylene glycol)	ZnO NPs Ag–ZnO NPs	Synthesized via chemical precipitation and reduction	length 183.3 nm width 66.7 nm	PPP/ZnO NPs PPP/Ag–ZnO NPs	Mixing in solution; solution casting technique	Powder aggregations or film	Non- specified
[73]	Catechol-terminated co-polymer (CCP)	Synthesized via RAFT polymerization	rGO AgNPs	rGO commercial; AgNP synthesized in situ	AgNPs 10 nm	CCP-rGO-Ag	GO decorated with CCP by mussel-inspired chemistry route; CCP-rGO covered with AgNPs via reduction	Powder	Non- specified
Zn-based NPs									
[74]	Co-polymers of poly(lactic-co-glycolic acid) PLGA	Commercial	ZnO NPs	Synthesized via laser ablation in liquid	45–60 nm	PLGA/ ZnO NPs	Obtained via low-temperature technology	Film	Prostheses; biomedical devices
[75]	Borosiloxane (Bs)	Obtained from PDMS and boric acid (BA) heating above 200 °C	ZnO NPs	Synthesized via laser ablation in liquid	90 nm	Bs/ ZnO NPs	Mixing in solution	Film	Prostheses and biomedical devices
[76]	Low-density polyethylene (LDPE); ethylene vinyl acetate (EVA)	Commercial	ZnO NPs	Synthesized via chemical precipitation	20–30 nm	LDPE/ PVA/ ZnO NPs	Mixing in solution using mono-screw extruder	Bulk composite	Non- specified
[77]	Chitosan (Cs); polyvinyl alcohol (PVA)	Commercial	ZnO NPs	Synthesized via sol–gel method	-	CS/PVA-nano ZnO	Mixing in solution; solution casting approach	Film	Non- specified
[78]	Poly(L-lactic acid) (PLLA)	Commercial	ZnCl_2_ NPs ZnO NPs	Synthesized via laser ablation in different liquids	30–70 nm	PLLA/ ZnCl_2_ NPs PLLA/ ZnO NPs	Mixing in solution; spin-coating	Nanosheets	Wound healing
[79]	Polymethylmethacrylate (PMMA)	Commercial	ZnO	Synthesized via “oxalate route” chemical process	-	PMMA/ ZnO	Mixing in solution; slurry composites pour into cylindrical molds	Orthoses	Orthotic devices
[80]	Polyvinyl alcohol (PVA)	Commercial	ZnO NPs	Synthesized in situ	50–150 nm	PVA/ ZnO NPs	ZnO chemically obtained in PVA solution; electrospinning	Nanofibers	Wound healing and tissue reconstruc- tion
[81]	Hydroxypropyl chitosan (HPCs); polyvinyl alcohol (PVA)	Commercial	ZnO-NPs	Commercial	30 nm	HPCs/ PVA/ ZnO-NPs	Mixing in solution	Hydrocolloid composite sponge	Wound- healing
[82]	Polycaprolactone (PCL)	Commercial	ZnO loaded with curcumin (ZC-NPs)	Commercial ZnO was loaded with curcumin	<100 nm	ZCPCL	Mixing in solution, electrospinning	Mats	Top-sheets in feminine sanitary hygiene napkins
[83]	Polycaprolactone (PCL)	Commercial	ZnO NPs (CA)	Commercial ZnO modified with citric acid monohydrate	20 to 40 nm 99.71 nm (ZnO-CA)	PCL/ZnO-CA NPs	Mixing in solution; solvent-casting process	Membrane	Non- specified
[15]	Polyvinylpyrrolidone (PVP)	Commercial	ZnO NPs ZnO-CTAB NPs ZnO-HTAB NPs	Commercial and chemically synthesized ZnO NPs were modified with Cetyltrimethylammonium bromide (CTAB) and Hexadecyltrimethylammonium bromide (HTAB)	-	PVP/ZnO NPs PVP/ZnO-CTAB NPs PVP/ZnO-HTAB NPs	Mixing in solution and centrifugally spun using centrifuge equipped with a spinneret	Nanofibers	Wound dressing
[84]	Chitin–cellulose (CH-CE)	Commercial	ZnO, CuO	Synthesized preliminary via sol–gel synthesis	15–23 nm	CH-CE@ZnO/CuO	Mixing in solution, solution cast method	Conductive nanocomposite films with adjusted semiconductors properties	Non- specified
[85]	Dental resin	Obtained from commercial Bis-GMA and TEGDMA via photopolymerization	Zn doped mesoporous silica nanoparticles (Zn-MSNs)	Synthesized via sol–gel method	about 138 nm	Zn-MSNs/Dental resin	Fillers mixed with resin	Dental resin	Dentistry
[86]	Acrylonitrile butadiene rubber (NBR)	Commercial	ZnO NRs; ZnO NPs; ZnO NPs with CaCO_3;_ ZnO NPs with TiO_2_	Commercial	3 μm 20 nm	NBR/ ZnO	Latex processing technique	Films	Medical devices
Cu-based NPs									
[87]	Polyvinyl pyrrolidone/ carboxymethyl cellulose (PVP/CMC)	Commercial	CuO NPs	Synthesized preliminary via laser ablation	<10 nm	CuONPs/PVP/ CMC	Mixing in solution	PVP/CMC polymer blend films modified with CuO nanoparticles	Coating
[20]	Polyvinyl alcohol (PVA); polyvinyl pyrrolidone (PVP)	Commercial	CuO NPs	Synthesized via laser ablation in situ	-	PVA/ PVP/CuO NPs	Pulsed laser ablation pf Cu target in PVA/PVP blend	Film	Wound healing
[88]	Chitosan (Cs)	Commercial	CuNPs	Commercial	~8.11 nm	Cs/ CuNPs	Mixing in solution; solution casting approach	Film	Non- specified
[89]	Polymethyl methacrylate (PMMA)	Commercial	CuFe_2_O_4_/Cu_2_O/CuO	Synthesized via electrical explosion of twisted copper and iron wires	10 to 100 nm	PMMA- CuFe_2_O_4_/ Cu_2_O/ CuO NPs	Mixing in solution; solution casting approach	Film	Non- specified
[90]	Polyaniline (PANI)	Synthesized by in situ oxidative polymerization	CuS NPs	Obtained via solvothermal synthesis	-	CuS@ PANI	PANI polymerized in the presence of CuS NPs	Core–shell particles	Non- specified
[91]	Isotactic polypropylene (iPP)	Commercial	CuNPs-PEI/GABA	CuNPs functionalized with polyethyleneimine and 4-aminobutyric acid	27.0 nm	iPP/ CuNPs	Mixing in solution	Plates	Non- specified
[17]	Polyvinyl alcohol (PVA)	Commercial	GO/L-Arg/CuO GO/L-Arg-Cu	GO prepared by Hammers’ method and modified with L-Arginine; Cu-containig particles synthsized via green synthesis	-	PVA/GO/L-Arg/ CuO PVA/GO/L-Arg-Cu	Mixing in solution, additional cross-linking by citric acid (CA)	Green cross-linked electrospun nanofber	Wound healing
[92]	Polypropylene (PP)	Commercial	Cu-TiO_2_	Obtained via soft-chemical hydrothermal technique	35 nm length 20 nm diameter	Cu-TiO_2_-PP	Mixing in solution; compressed by compression molding machine	Film	Non- specified
[35]	Polyvinyl alcohol (PVA); tannic acid (TA)	Commercial	CuO/SiO_2_	Simultaneously synthesized via chemical approach	Between 50 and 90 nm	PVA/TA/CuO/SiO_2_	Mixing in solution	Biofilm	Rapid wound dressing
TiO_2_ NPs									
[9]	Polyvinyl alcohol (PVA); sodium alginate (SA)	Commercial	TiO_2_ NPs	Green synthesis using Aloe vera leaves extract	-	PVA/SA/TiO_2_ NPs	Mixing in solution; solvent-casting process	Film	Wound dressing
[93]	Poly(3-hydroxybuty-rate-co-3-hydroxyvalerate) (PHBV)	Commercial	TiO_2_ NPs	Commercial	-	PHBV/ TiO_2_ NPs	Mixing in solution, electrospinning	Nanofiber scaffolds	Non- specified
[94]	Poly(ethylene oxide) (PEO); polyvinylpyrrolidone (PVP)	Commercial	TiO_2_ NPs	Chemically synthesized	12.1 to 19.19 nm	PEO/PVP/TiO_2_ NPs	Mixing in solution; solvent-casting process	Film	Non- specified
[16]	Polyvinyl alcohol (PVA); sodium alginate (SA); sodium carboxymethyl cellulose (CMC-Na); waterborne polyurethane (WPU); polyvinylpyrrolidone (PVP)	Commercial components were blended in water	nTiO_2_	Commercial	20–30 nm	PVA/SA/CMC/ WPU/ PVP/ nTiO_2_	Mixing in solution	Transparent protective gel membrane	Wound protection
[95]	Polycaprolactone (PCL)	Commercial	TiO_2_ modified with poly-ortho-toluidine (POT)	Commercial TiO_2_ modified with POT via oxidative polymerization	-	POT- TiO_2_/PCL	Mixing in solution, solution cast method	Film	Tissue engineering
[96]	Polystyrene (PS); poly(vinyl alcohol) (PVA)	PS synthesized in situ; PVA commercial	Vinyltriethoxysilane (VTES) modified TiO_2_	VTES and TiO_2_ commercial	30 nm	V-TiO_2_/ PS/PVA	PS polymerized in the presence of V-TiO_2_; PVA solution was added	Microspheres	Hemoperfusion adsorbent for efficient bilirubin removal
[97]	High-density polyethylene (HDPE)	Commercial	TiO_2_ hydroxyapatite (HAp)	Commercial with additional treatment	10–14 nm	HDPE/ HAp/ TiO_2_	Powders mixing and melting; compaction technique	Composite sheets	Non- specified
Other NPs									
[98]	Thermoplastic elastomers TPE88 (TPE)	Commercial	Hydrophobic carbon quantum dots (hCQDs)	Chemical synthesis using polymerization	-	TPE/ hCQDs	TPE filament 3D printed and immersed into hCQDs solution	Filament	Medical tools and devices
[99]	Polycaprolactone (PCL)	Commercial	Graphene oxide (GO); CeO_2_	GO synthesized via modified Hummer’s technique; CeO_2_ obtained via hydrothermal method	-	CL-GO- CeO_2_	Mixing in solution; solvent-casting process	Film	Bone tissue engineering
[100]	Chitosan (Ch)	Commercial	SPION	Co-precipitation in situ using microfluidic reactor	6.8 ± 0.6 nm	Ch- SPION	Composite obtained using microfluidic reactor	Powder	MRI imaging
[101]	Sodium alginate-containing curcumin	Commercial	Fe_3_O_4_NPs	Synthesized via co-precipitation	390±14.76 nm (DLS) 359 nm (SEM)	SA- Curcumin/Fe_3_O_4_ NPs	Mixing in solution	Particles	Drug delivery
[102]	Poly(L-lactide) (PLLA)	Commercial	MgO NP surface modified by FeCl_3_ and tannic acid (TA) (FeCl_3_–TA/MgO)	Commercial MgO modified with commercial FeCl_3_ and TA	20 nm	PLLA/ FeCl_3_– TA/MgO	Mixing in solution; 4D processing	Porous scaffolds	Bone tissue engineering
[103]	Dental resin of triethylene glycol dimethacrylate (TEGDMA), polymethyl methacrylate (PMMA), acrylic matrix	Solvent evaporation method	MPS-grafted SiO_2_ NPs	Commercial SiO_2_ modified with MPS (3-methacryloxypropyl trimethoxysilane)	15–20 nm specific surface area of 210–240 m^2^/g	MPS- SiO_2_NPs/ Dental resin	Mixing and photo initiated polymerization	Self-healing dental composite	Dentistry
[104]	Dental resin	Obtained from Bis-GMA and TEGDMA	core–shell chlorhexidine/amorphous calcium phosphate (CHX/ACP) NPs silanization silica fillers	Synthesized via vesicle templating technology	98.48 ± 17.61 nm	Dental resin/ CHX/ ACP/ SiO_2_	Fillers mixed evenly with pure resin	Composite resin	Dentistry
[105]	Microcapsules (MCs) contained: poly(urea-formaldehyde) (PUF) shells; N, N-dihydroxyethyl-p-toluidine (DHEPT); triethylene glycol dimethacrylate (TEGDMA)	Synthesized in situ	Nanoantibacterial inorganic fillers (NIFs): nanosilica; quaternary ammonium salts (QAS)	QAS chemically synthesized and conjugated with SiO_2_	-	MCs/ NIFs	In situ polymerization and liquid-phase precipitation	Microcapsules for self-healing of dental resin	Dentistry
[106]	Dental resin matrix	Obtained from commercial Bis-GMA and TEGDMA via photopolymerization	MgONPs SiO_2_	Commercial	∼50 nm ∼2 μm	Bis-GMA/TEGDMA/MgONPs/SiO_2_	Mixing under yellow light	Photoactivating paste dental resin composite	Repairing dental defects
[107]	Sodium alginate (ALG)	Polymerized in situ	CIP-loaded nanohydroxyapatite (HANPs)	Synthesized and loaded with CIP	length 52 nmdiameter 8 nm	CIP- HANPs/ALG	HANPs added to ALG solution as cross-linking agent	Powder	Bone filler
[108]	Sodium alginate (Na-ALG)/polyvinyl alcohol (PVA)	Commercial	Mesoporous bioactive glass NPs doped with Ag and Cu	Synthesized preliminary via Stöber process (sol–gel synthesis)	-	Na-ALG/PVA/Cu–Ag MBGNs	Mixing in solution, 3D printing	Skin scaffolds	Patient- specific implants
[109]	Poly (xylitol maleate adipate) (PXMA)	Synthesized by melt polycondensation without catalyst	Nanobioactive glass (n-BG) SiO_2_–CaO–P_2_O_5_	Synthesized via sol–gel method	13–24 nm	n-BG/ PXMA	Mixing in solution	Film	Bone construction
[36]	Carrageenan (CRG)	Commercial	CeO_2_ NPs	Commercial	15–30 nm	CRG- CeO_2_	Mixing in solution with glutaraldehyde	Sponge	Wound healing, blood clotting agent
[11]	Cellulose acetate (CelAc); chitosan (Chit)	Commercial	CeO_2_	Commercial	<25 nm	CelAc/Chit/ CeO_2_	Mixing in solution	Film	Wound dressing
[110]	Borsiloxane (BS)	Obtained from hydroxyl-terminated polydimethylsiloxane (PDMS) and boric acid (BA) heating above 200 °C	Al_2_O_3_	Synthesized preliminary via laser ablation in liquid	Average hydrodynamic diameter ~45 nm	Al_2_O_3_NPs/BS	Mixing in solution	Films	For prostheses and biomedical devices
[111]	Chitosan	Commercial	CoO	Commercial	-	CS-CoO	Solution casting approach	Film	Potential pharma- ceutical applications
[30]	Polylactic acid (PLA)	Commercial	Montmorillonite (MMT) NPswith adsorbed B compounds	Commercial boron nitride (BN), zinc borate (ZB), phenylboronic acid (PBA), and MMT mixed	200–300 nm	MMT/Bs/PLA	Mixing in solution, electrospinning	Micron/submicron fibers	Wound dressing
[112]	Vinylidene fluoride-co-hexafluoropropylene (PVDF-HFP); microcrystalline cellulose (MCC)	Commercial	LiFe_5_O_8_ (LFO)	Chemical synthesis	-	PVDF- HFP/ MCC/ LFO	Mixing in solution, electrospinning	Fiber mats	Coating
[33]	PDA-modifed poly(lactic-co-glycolic) acid (PLGA)	Polymerization of dopamine on the PLGA membrane (commercial)	AuNPs; antibacterial peptides (Os)	AuNPs synthesized in situ via chemical reduction; Os commercial	-	Os/ Au-PDA@PLGA	Sequential immersion of membrane into solution for Au reduction and for Os attachment	Membrane	Wound repair
[3]	Silk fibroin (SF)and collagen (CL)	Silk fibroin obtained via extraction; collagen commercial	Pd Pt	Synthesized in situ via chemical reduction	8.64 nm 7.36 nm	SF/CL/ Pd–Pt	Mixing in solution, electrospinning	Composite nanofber scafolds	Wound dressing
[8]	Polycaprolactone (PCL); chitosan (CHT)	Commercial	Bentonite (Ben)	Commercial	-	PCL/ CHT/Ben	Mixing in solution, electrospinning	3D scaffolds	Wound dressing
[113]	Cissus quadrangularis fiber (CQF); bisphenol F type LY556 epoxy resin; Araldite HY951 hardener	Commercial	CaO NPs	Commercial	-	Epoxy resin/CaO	Mixing, compressing	Laminate	Non- specified
[114]	Low-density polyethylene (LDPE)	Commercial	CaO NPs CaO-OA	CaO chemically synthesized and modified with oleic acid (OA)	25 and 55 nm	LDPE/ CaO NPs	Mixing in nitrogen atmosphere and press-molded at 170 °C	Bulk composite	Non- specified
[115]	Chitosan (CS)	Commercial	Te NPs	Synthesized in situ	37.48 ± 14.56 nm	CS-Te NPs	Te NPs obtained in the presence of CS	Particles	Non- specified
[116]	Cellulose	Commercial	NiO NPs	Synthesized in situ	20–30 nm	Cellulose/NiO NPs	NiO chemically synthesized in the presence of cellulose	Powder	Treatment of visceral leishma- niasis

**Table 2:** General properties of polymers.

Polymer	State	Toxicity	Cost	Hydrophilicity	Solubility in Water	Mechanic Characteristics	Stability
PVA	Semi- crystalline	Non-toxic	Low cost	Hydrophilic	Soluble in hot water	Superior mechanical properties; high tensile strength and flexibility	Low thermal stability
Cs	Semi- crystalline	Non-toxic	Expensive	Hydrophilic and hydrophobic	Water- soluble in acidic media	Limited mechanical and barrier properties; low strength	Poor stability
CE	Semi- crystalline	Non-toxic	Low cost	Extensively hydrophilic	Insoluble in water	Superior mechanical properties; low flexibility	Sustainable
PCL	Semi- crystalline	Non-toxic	Commercially available	Hydrophobic	Long-term degradation in the presence of water	Good mechanical properties; flexible; tough	Good thermal stability
PLA	Amorphous; semi-crystalline; highly crystalline	Non-toxic	High cost	Hydrophobic	Insoluble in water	High strength but brittle; poor toughness; shape-memory effect	Relatively low thermal stability. Chemically inert
PVP	Amorphous	Low-toxic	Low cost	Hydrophilic and hydrophobic	Water-soluble	Poor mechanical stability	Chemically inert, temperature-resistant, pH-stable
SA	Amorphous; semi- crystalline	Low-toxic	Relatively low cost	Hydrophilic	Outstan- ding water absorption and holding ability	Poor mechanical strength	Relatively low stability. pH- sensitive
PMMA	Amorphous	Non-toxic	Low cost of production	Hydrophobic	Insoluble in water	Good mechanical properties; strong, tough, and lightweight	Good physical, chemical and thermal stability; superior environmental stability
PDA		Non-toxic	High-cost	Moderately hydrophilic	Water- soluble		Poor thermal stability; durable
PEO	Semi- crystalline	Non-toxic	Low-cost	Hydrophilic	Water- soluble	Strong mechanical properties; strength and resistance to impact and stress	High chemical and electrochemical stability; thermal stability; durability
PP	Increased crystallinity	Generally considered safe for use	Comparatively low cost	Hydrophobic	Insoluble in water	Satisfactory mechanical strength; flexible material; outstanding fatigue resistance	High thermal and chemical stability; superior chemical resistance

**Table 3:** Properties of polymers specific to their use for biomedical applications.

Polymer	Degradability	Biocompatibility	Bioactivity	Surface Groups	Other
PVA	Biodegradable under both aerobic and anaerobic conditions	Biocompatible			Synthetic polymer; transparent
Cs	Biodegradable	Exceptional biocompatibility	Unique bioactivity; Antibacterial and antioxidant properties; bioadhesivity	Reactive groups	Natural origination; semi-synthetic polymer
CE	Biodegradable	Biocompatible		Hydroxyl groups	Renewable polymer; natural origination
PCL	Biodegradable	Excellent biocompatibility	Non-immunogenic		Synthetic polymer; low melting point; good blend ability; easy to functionalize
PLA	Slow biodegradation rate	Biocompatible	Low cell adhesion	Lack of reactive side-chain groups	Can be synthesized from renewable resources; good layer adhesion. good printability; widely used in 3D printing
PVP	Biodegradable	Excellent biocompatibility	Inert; excreted rapidly and completely through kidney	Hydrophilic and hydrophobic functional groups	Synthetic polymer; film-forming capability; great stabilizer; protective agent; good electrical properties; good transparency; adhesive properties; undergoes cross-linking
SA	Good biodegradability	Good biocompatibility	Well tolerated by the immune system	Abundant hydroxyl and carboxyl groups	Natural origination; renewable; excellent gelling and thickening properties
PMMA	Non-degradable	Good biocompatibility		Easily hydrolyzed ester groups	Synthetic polymer; malleability; transmits up to 92% of visible light
PDA	Great biodegradability		Promotes cell adhesion and proliferation on substrates; promotes adhesion of implants onto tissue; stimulates bone formation around the implant; scavenges harmful ROS	Abundant functional groups; large volumes of catechol, quinine, and amine groups	Synthetic eumelanin polymer; coating material; functionalizing and modifying agent; redox activities; photoprotective and superparamagnetic properties
PEO	Resistant to degradation	Excellent biocompatibility	Minimal health risks		Synthetic polymer; film-forming capability; high ionic conductivity
PP	Non-biodegradable	Biocompatible			Synthetic polymer; thermoplastic; Recyclable; superior electrical insulation; transparent

## Data Availability

No new data were created or analyzed in this study.

## References

[1] Ghazzy A., Naik R.R., Shakya A.K. (2023). Metal–Polymer Nanocomposites: A Promising Approach to Antibacterial Materials. Polymers.

[2] Chang T.-K., Cheng T.-M., Chu H.-L., Tan S.-H., Kuo J.-C., Hsu P.-H., Su C.-Y., Chen H.-M., Lee C.-M., Kuo T.-R. (2019). Metabolic Mechanism Investigation of Antibacterial Active Cysteine-Conjugated Gold Nanoclusters in Escherichia coli. ACS Sustain. Chem. Eng..

[3] Arumugam M., Murugesan B., Chinnalagu D., Balasekar P., Cai Y., Sivakumar P.M., Rengasamy G., Chinniah K., Mahalingam S. (2024). Electrospun Silk Fibroin and Collagen Composite Nanofiber Incorporated with Palladium and Platinum Nanoparticles for Wound Dressing Applications. J. Polym. Environ..

[4] Balcucho J., Narváez D.M., Tarazona N.A., Castro-Mayorga J.L. (2023). Microbially Synthesized Polymer-Metal Nanoparticles Composites as Promising Wound Dressings to Overcome Methicillin-Resistance Staphylococcus aureus Infections. Polymers.

[5] Chen J., Fan L., Yang C., Wang S., Zhang M., Xu J., Luo S. (2020). Facile synthesis of Ag nanoparticles-loaded chitosan antibacterial nanocomposite and its application in polypropylene. Int. J. Biol. Macromol..

[6] Cobos M., De-La-Pinta I., Quindós G., Fernández M., Fernández M. (2020). Synthesis, Physical, Mechanical and Antibacterial Properties of Nanocomposites Based on Poly(vinyl alcohol)/Graphene Oxide–Silver Nanoparticles. Polymers.

[7] Elabbasy M.T., Algahtani F.D., Othman M.S., Ahmad K., Maysara S., Al-Najjar M.A.A., El-Morsy M.A., Menazea A.A. (2024). Laser deposited ultra-thin silver nanoparticles on CMC-PVA blend film as sheet for wound dressings. Mater. Chem. Phys..

[8] Hashemi S.S., Mohammadi A.A., Kian M., Rafati A., Ghaedi M., Ghafari B. (2024). Fabrication and evaluation of the regenerative effect of a polycaprolactone/chitosan nanofibrous scaffold containing bentonite nanoparticles in a rat model of deep second-degree burn injury. Iran. J. Basic Med. Sci..

[9] Ibrahim M.A., Nasr G.M., Ahmed R.M., Kelany N.A. (2024). Physical characterization, biocompatibility, and antimicrobial activity of polyvinyl alcohol/sodium alginate blend doped with TiO_2_ nanoparticles for wound dressing applications. Sci. Rep..

[10] Iswarya S., Bharathi M., Hariram N., Theivasanthi T., Gopinath S.C.B. (2024). Solid polymer electrolyte and antimicrobial performance of Polyvinyl alcohol/Silver nanoparticles composite film. Results Chem..

[11] Kalaycıoğlu Z., Kahya N., Adımcılar V., Kaygusuz H., Torlak E., Akın-Evingür G., Erim F.B. (2020). Antibacterial nano cerium oxide/chitosan/cellulose acetate composite films as potential wound dressing. Eur. Polym. J..

[12] Karami R., Moradipour P., Arkan E., Zarghami R., Rashidi K., Darvishi E. (2023). Biocompatible nano-bandage modified with silver nanoparticles based on herbal for burn treatment. Polym. Bull..

[13] Khaje K., Ghaee A., Ghaie F., Hosseini I., Seifi S. (2024). Gelatin-based scaffold incorporating Ag nanoparticles decorated polydopamine nanoparticles for skin tissue engineering. Int. J. Polym. Mater. Polym. Biomater..

[14] Kumar M., Dhiman S.K., Bhat R., Saran S. (2023). In situ green synthesis of AgNPs in bacterial cellulose membranes and antibacterial properties of the composites against pathogenic bacteria. Polym. Bull..

[15] Sanchez J.A., Ibrahim A., Materon L., Parsons J.G., Alcoutlabi M. (2023). Centrifugally spun PVP/ZnO composite fibers with different surfactants and their use as antibacterial agents. J. Appl. Polym. Sci..

[16] Shao K., Li X., Tan B., Wang Q., Guo Z., Liu T., Dong L. (2023). Polyvinyl alcohol composite gel membrane modified by natural polysaccharide and waterborne polyurethane. Polym. Eng. Sci..

[17] Amoee S., Monad N., Hamidinezhad H., Karimian M. (2024). Fabrication and investigation antibacterial properties of green cross-linked PVA/GO/L-Arg-Cu electrospun nanofiber. Polym. Bull..

[18] Bagheri M., Validi M., Gholipour A., Makvandi P., Sharifi E. (2021). Chitosan nanofiber biocomposites for potential wound healing applications: Antioxidant activity with synergic antibacterial effect. Bioeng. Transl. Med..

[19] Abd El-Kader M.F.H., Elabbasy M.T., Ahmed M.K., Menazea A.A. (2021). Structural, morphological features, and antibacterial behavior of PVA/PVP polymeric blends doped with silver nanoparticles via pulsed laser ablation. J. Mater. Res. Technol..

[20] El-Kader M.F.H.A., Elabbasy M.T., Adeboye A.A., Menazea A.A. (2021). Nanocomposite of PVA/PVP blend incorporated by copper oxide nanoparticles via nanosecond laser ablation for antibacterial activity enhancement. Polym. Bull..

[21] Guo C., Cheng F., Liang G., Zhang S., Jia Q., He L., Duan S., Fu Y., Zhang Z., Du M. (2022). Copper-based polymer-metal–organic framework embedded with Ag nanoparticles: Long-acting and intelligent antibacterial activity and accelerated wound healing. Chem. Eng. J..

[22] Korniienko V., Husak Y., Diedkova K., Varava Y., Grebnevs V., Pogorielova O., Bērtiņš M., Korniienko V., Zandersone B., Ramanaviciene A. (2024). Antibacterial Potential and Biocompatibility of Chitosan/Polycaprolactone Nanofibrous Membranes Incorporated with Silver Nanoparticles. Polymers.

[23] Li J., Xu X., Ma X., Cui M., Wang X., Chen J., Zhu J., Chen J. (2024). Antimicrobial Nonisocyanate Polyurethane Foam Derived from Lignin for Wound Healing. ACS Appl. Bio. Mater..

[24] Luo H., Huang T., Li X., Wang J., Lv T., Tan W., Gao F., Zhang J., Zhou B. (2022). Synergistic antibacterial and wound-healing applications of an imidazole-based porous organic polymer encapsulated silver nanoparticles composite. Microporous Mesoporous Mater..

[25] Zhang L., Yu Y., Zheng S., Zhong L., Xue J. (2021). Preparation and properties of conductive bacterial cellulose-based graphene oxide-silver nanoparticles antibacterial dressing. Carbohydr. Polym..

[26] Zhang Z., Shi L., Chu L., Chen P., Sun P., Chen Z., Wei L., Zhou B. (2023). Crown ether-based porous organic polymer encapsulated Ag2[Fe(CN)5NO] composite towards ultra-low dose efficient sterilization and wound healing application. Mater. Today Chem..

[27] Zhang F., Yao Q., Niu Y., Chen X., Zhou H., Bai L., Kong Z., Li Y., Cheng H. (2024). In Situ Fabrication of Silver Nanoparticle-Decorated Polymeric Vesicles for Antibacterial Applications. Chem. Open.

[28] Jia H., Zeng X., Fan S., Cai R., Wang Z., Yuan Y., Yue T. (2022). Silver nanoparticles anchored magnetic self-assembled carboxymethyl cellulose-ε-polylysine hybrids with synergetic antibacterial activity for wound infection therapy. Int. J. Biol. Macromol..

[29] Zhu G., Sun Z., Hui P., Chen W., Jiang X. (2020). Composite Film with Antibacterial Gold Nanoparticles and Silk Fibroin for Treating Multidrug-Resistant E. coli-Infected Wounds. ACS Biomater. Sci. Eng..

[30] Dilmani S.A., Koç S., Erkut T.S., Gümüşderelioğlu M. (2024). Polymer-clay nanofibrous wound dressing materials containing different boron compounds. J. Trace Elem. Med. Biol..

[31] Wang B., Wang H., Wang Z., Tang J., Yuan X., Zhang Y., Chen H., Yu W., Song M. (2023). Preparation of AgBrNPs@copolymer-decorated chitosan with synergistic antibacterial activity. Mater. Today Commun..

[32] Guimarães M.L., da Silva F.A.G., da Costa M.M., de Oliveira H.P. (2022). Coating of conducting polymer-silver nanoparticles for antibacterial protection of Nile tilapia skin xenografts. Synth. Met..

[33] Dong Y., Wang Z., Wang J., Sun X., Yang X., Liu G. (2024). Mussel-inspired electroactive, antibacterial and antioxidative composite membranes with incorporation of gold nanoparticles and antibacterial peptides for enhancing skin wound healing. J. Biol. Eng..

[34] Chen M., Ma C., Zhou C., Li Z., Li R. (2023). Preparation and Properties of Water-Resistant Antibacterial Curcumin/Silver Composite Nanofiber. Fibers Polym..

[35] Miralaei N., Mohammadimehr M., Farazin A., Ghasemi A.H., Bargozini F. (2023). Design, fabrication, evaluation, and in vitro study of green biomaterial and antibacterial polymeric biofilms of polyvinyl alcohol/tannic acid/CuO/SiO2. J. Mech. Behav. Biomed. Mater..

[36] Alizadeh K., Dezvare Y., Kamyab S., Amirian J., Brangule A., Bandere D. (2023). Development of Composite Sponge Scaffolds Based on Carrageenan (CRG) and Cerium Oxide Nanoparticles (CeO2 NPs) for Hemostatic Applications. Biomimetics.

[37] Asha A.B., Ounkaew A., Peng Y.-Y., Gholipour M.R., Ishihara K., Liu Y., Narain R. (2023). Bioinspired antifouling and antibacterial polymer coating with intrinsic self-healing property. Biomater. Sci..

[38] Chen K., Wang F., Liu S., Wu X., Xu L., Zhang D. (2020). In situ reduction of silver nanoparticles by sodium alginate to obtain silver-loaded composite wound dressing with enhanced mechanical and antimicrobial property. Int. J. Biol. Macromol..

[39] Chen Z., Sun J., Zhan Z., Yuan Y., Tian X., Jin J., Wu W., Ayikanbaier K. (2024). Multifunctional flexible strain sensor based on three-dimensional core-shell structures of silver nanoparticles/natural rubber. J. Appl. Polym. Sci..

[40] Haladu S.A., Elsayed K.A., Olanrewaju Alade I., Alheshibri M., Al Baroot A., Ali S.A., Kotb E., Manda A.A., Ul-Hamid A., Dafalla H.D.M. (2022). Laser-assisted fabrication of silver quantum dots/polyaspartate polymer composite for antimicrobial applications. Opt. Laser Technol..

[41] Li H., Luo R., Qu J. (2023). Poly(methyl methacrylate-co-butyl acrylate) copolymer/Ag nanocomposites prepared by latex mixing for multifunctional coatings. Polym. Compos..

[42] Liu F., Cheng X., Xiao L., Wang Q., Yan K., Su Z., Wang L., Ma C., Wang Y. (2021). Inside-outside Ag nanoparticles-loaded polylactic acid electrospun fiber for long-term antibacterial and bone regeneration. Int. J. Biol. Macromol..

[43] Quintero-Quiroz C., Botero L.E., Zárate-Triviño D., Acevedo-Yepes N., Escobar J.S., Pérez V.Z., Cruz Riano L.J. (2020). Synthesis and characterization of a silver nanoparticle-containing polymer composite with antimicrobial abilities for application in prosthetic and orthotic devices. Biomater. Res..

[44] Ragab H.M., Diab N.S., AlElaimi M., Alghamdi A.M., Farea M.O., Farea A. (2024). Fabrication and Characterization of Silver Nanoparticle-Doped Chitosan/Carboxymethyl Cellulose Nanocomposites for Optoelectronic and Biological Applications. ACS Omega.

[45] Sun D., Turner J., Jiang N., Zhu S., Zhang L., Falzon B.G., McCoy C.P., Maguire P., Mariotti D., Sun D. (2020). Atmospheric pressure microplasma for antibacterial silver nanoparticle/chitosan nanocomposites with tailored properties. Compos. Sci. Technol..

[46] Teper P., Oleszko-Torbus N., Bochenek M., Hajduk B., Kubacki J., Jałowiecki Ł., Płaza G., Kowalczuk A., Mendrek B. (2022). Hybrid nanolayers of star polymers and silver nanoparticles with antibacterial activity. Colloids Surf. B Biointerfaces.

[47] Vidakis N., Michailidis N., David C., Papadakis V., Argyros A., Sagris D., Spiridaki M., Mountakis N., Nasikas N.K., Petousis M. (2024). Polyvinyl alcohol as a reduction agent in material extrusion additive manufacturing for the development of pharmaceutical-grade polypropylene/silver nanocomposites with antibacterial properties. Mater. Today Commun..

[48] Wang B., Guo W., Liu X., He Y., Song P., Wang R. (2020). Fabrication of silver-decorated popcorn-like polymeric nanoparticles for enhanced antibacterial activity. Appl. Surf. Sci..

[49] Wang B.-B., Quan Y.-H., Xu Z.-M., Zhao Q. (2020). Preparation of highly effective antibacterial coating with polydopamine/chitosan/silver nanoparticles via simple immersion. Prog. Org. Coat..

[50] Wang X., Yan H., Hang R., Shi H., Wang L., Ma J., Liu X., Yao X. (2021). Enhanced anticorrosive and antibacterial performances of silver nanoparticles/polyethyleneimine/MAO composite coating on magnesium alloys. J. Mater. Res. Technol..

[51] Wang J.-L., Wang S., Zhong P., Chen Y., Zhao Z., Liu W. (2024). Multimechanism Long-Term Antibacterial Coating with Simultaneous Antifouling, Contact-Active, and Release-Kill Properties. ACS Appl. Polym. Mater..

[52] Xiong C., Wang T., Zhang Y., Duan C., Zhang Z., Zhou Q., Xiong Q., Zhao M., Wang B., Ni Y. (2023). Multifunctional Conductive Material Based on Intelligent Porous Paper Used in Conjunction with a Vitrimer for Electromagnetic Shielding, Sensing, Joule Heating, and Antibacterial Properties. ACS Appl. Mater. Interfaces.

[53] Smirnova V.V., Chausov D.N., Serov D.A., Kozlov V.A., Ivashkin P.I., Pishchalnikov R.Y., Uvarov O.V., Vedunova M.V., Semenova A.A., Lisitsyn A.B. (2021). A Novel Biodegradable Composite Polymer Material Based on PLGA and Silver Oxide Nanoparticles with Unique Physicochemical Properties and Biocompatibility with Mammalian Cells. Materials.

[54] Zhao X., Kang L., Zhang B., Liu L., Sun H., Shen X., Su F., Li S. (2023). Biodegradable nanocomposites prepared from poly(butylene adipate terephthalate) and silver nanoparticles for applications as disposable medical equipments. Polym. Adv. Technol..

[55] Arif W., Rana N., Saleem I., Tanweer T., Khan M., Alshareef S., Sheikh H., Alaryani F., Al-Kattan M., Alatawi H. (2022). Antibacterial Activity of Dental Composite with Ciprofloxacin Loaded Silver Nanoparticles. Molecules.

[56] Cao B., Zhang J., Ma Y., Wang Y., Li Y., Wang R., Cao D., Yang Y., Zhang R. (2024). Dual-Polymer Functionalized Melanin-AgNPs Nanocomposite with Hydroxyapatite Binding Ability to Penetrate and Retain in Biofilm Sequentially Treating Periodontitis. Small.

[57] Datta L.P., Dutta D., Mukherjee R., Das T.K., Biswas S. (2024). Polyoxometalate-Polymer Directed Macromolecular Architectonics of Silver Nanoparticles as Effective Antimicrobials. Chem. Asian J..

[58] Mogharbel A.T., Ibarhiam S.F., Alqahtani A.M., Attar R.M.S., Alshammari K.F., Bamaga M.A., Al-Qahtani S.D., El-Metwaly N.M. (2023). Plasma-assisted in-situ preparation of silver nanoparticles and polypyrrole toward superhydrophobic, antimicrobial and electrically conductive nonwoven fabrics from recycled polyester waste. J. Ind. Eng. Chem..

[59] Abdelaziz D., Hefnawy A., Al-Wakeel E., El-Fallal A., El-Sherbiny I.M. (2021). New biodegradable nanoparticles-in-nanofibers based membranes for guided periodontal tissue and bone regeneration with enhanced antibacterial activity. J. Adv. Res..

[60] Guo W., Liu W., Xu L., Feng P., Zhang Y., Yang W., Shuai C. (2020). Halloysite nanotubes loaded with nano silver for the sustained-release of antibacterial polymer nanocomposite scaffolds. J. Mater. Sci. Technol..

[61] Huang X., Ge M., Wang H., Liang H., Meng N., Zhou N. (2022). Functional modification of polydimethylsiloxane nanocomposite with silver nanoparticles-based montmorillonite for antibacterial applications. Colloids Surf. A Physicochem. Eng. Asp..

[62] Jatoi A.W., Kim I.S., Ni Q.-Q. (2019). Cellulose acetate nanofibers embedded with AgNPs anchored TiO2 nanoparticles for long term excellent antibacterial applications. Carbohydr. Polym..

[63] Jatoi A.W., Ogasawara H., Kim I.S., Ni Q.-Q. (2020). Cellulose acetate/multi-wall carbon nanotube/Ag nanofiber composite for antibacterial applications. Mater. Sci. Eng. C.

[64] Khan M.U.A., Abd Razak S.I., Mehboob H., Abdul Kadir M.R., Anand T.J.S., Inam F., Shah S.A., Abdel-Haliem M.E.F., Amin R. (2021). Synthesis and Characterization of Silver-Coated Polymeric Scaffolds for Bone Tissue Engineering: Antibacterial and In Vitro Evaluation of Cytotoxicity and Biocompatibility. ACS Omega.

[65] Lipovka A., Fatkullin M., Shchadenko S., Petrov I., Chernova A., Plotnikov E., Menzelintsev V., Li S., Qiu L., Cheng C. (2023). Textile Electronics with Laser-Induced Graphene/Polymer Hybrid Fibers. ACS Appl. Mater. Interfaces.

[66] Liu C., Ling J., Yang L.-Y., Ouyang X.-k., Wang N. (2023). Chitosan-based carbon nitride-polydopamine-silver composite dressing with antibacterial properties for wound healing. Carbohydr. Polym..

[67] Sarıipek F.B., Sevgi F., Dursun S. (2022). Preparation of poly(ε-caprolactone) nanofibrous mats incorporating graphene oxide-silver nanoparticle hybrid composite by electrospinning method for potential antibacterial applications. Colloids Surf. A Physicochem. Eng. Asp..

[68] Shuai C., Xu Y., Feng P., Wang G., Xiong S., Peng S. (2019). Antibacterial polymer scaffold based on mesoporous bioactive glass loaded with in situ grown silver. Chem. Eng. J..

[69] Shuai C., Liu G., Yang Y., Qi F., Peng S., Yang W., He C., Wang G., Qian G. (2020). A strawberry-like Ag-decorated barium titanate enhances piezoelectric and antibacterial activities of polymer scaffold. Nano Energy.

[70] Su X., Zhai Y., Jia C., Xu Z., Luo D., Pan Z., Xiang H., Yu S., Zhu L., Zhu M. (2023). Improved Antibacterial Properties of Polylactic Acid-Based Nanofibers Loaded with ZnO–Ag Nanoparticles through Pore Engineering. ACS Appl. Mater. Interfaces.

[71] Wang B., Moon J.R., Ryu S., Park K.D., Kim J.H. (2020). Antibacterial 3D graphene composite gel with polyaspartamide and tannic acid containing in situ generated Ag nanoparticle. Polym. Compos..

[72] Zhong Q., Long H., Hu W., Shi L., Zan F., Xiao M., Tan S., Ke Y., Wu G., Chen H. (2020). Dual-Function Antibacterial Micelle via Self-Assembling Block Copolymers with Various Antibacterial Nanoparticles. ACS Omega.

[73] Zhou S., Ji H., Liu L., Feng S., Fu Y., Yang Y., Lü C. (2020). Mussel-inspired coordination functional polymer brushes-decorated rGO-stabilized silver nanoparticles composite for antibacterial application. Polym. Chem..

[74] Burmistrov D.E., Simakin A.V., Smirnova V.V., Uvarov O.V., Ivashkin P.I., Kucherov R.N., Ivanov V.E., Bruskov V.I., Sevostyanov M.A., Baikin A.S. (2021). Bacteriostatic and Cytotoxic Properties of Composite Material Based on ZnO Nanoparticles in PLGA Obtained by Low Temperature Method. Polymers.

[75] Chausov D.N., Burmistrov D.E., Kurilov A.D., Bunkin N.F., Astashev M.E., Simakin A.V., Vedunova M.V., Gudkov S.V. (2021). New Organosilicon Composite Based on Borosiloxane and Zinc Oxide Nanoparticles Inhibits Bacterial Growth, but Does Not Have a Toxic Effect on the Development of Animal Eukaryotic Cells. Materials.

[76] Galli R., Hall M.C., Breitenbach E.R., Colpani G.L., Zanetti M., de Mello J.M.M., Silva L.L., Fiori M.A. (2020). Antibacterial polyethylene–Ethylene vinyl acetate polymeric blend by incorporation of zinc oxide nanoparticles. Polym. Test..

[77] Hezma A.M., Rajeh A., Mannaa M.A. (2019). An insight into the effect of zinc oxide nanoparticles on the structural, thermal, mechanical properties and antimicrobial activity of Cs/PVA composite. Colloids Surf. A Physicochem. Eng. Asp..

[78] Ishak M.Q.H., Shankar P., Turabayev M.E., Kondo T., Honda M., Gurbatov S.O., Okamura Y., Iwamori S., Kulinich S.A. (2022). Biodegradable Polymer Nanosheets Incorporated with Zn-Containing Nanoparticles for Biomedical Applications. Materials.

[79] Khlifi K., Atallah M.S., Cherif I., Karkouch I., Barhoumi N., Attia-Essaies S. (2023). Synthesis of ZnO nanoparticles and study of their influence on the mechanical properties and antibacterial activity of PMMA/ZnO composite for orthotic devices. Surf. Interfaces.

[80] Norouzi M.A., Montazer M., Harifi T., Karimi P. (2021). Flower buds like PVA/ZnO composite nanofibers assembly: Antibacterial, in vivo wound healing, cytotoxicity and histological studies. Polym. Test..

[81] Wang Q., Zhang X., Fang X., Sun L., Wang X., Chen H., Zhu N. (2023). Antimicrobial hydrocolloid composite sponge with on-demand dissolving property, consisting mainly of zinc oxide nanoparticles, hydroxypropyl chitosan, and polyvinyl alcohol. J. Polym. Eng..

[82] Banerjee A., Roy P., Chakraborty J., Majumder M. (2024). Environmentally degradable curcumin/zinc oxide nanoparticles-incorporated polycaprolactone films for use as top-sheets in feminine sanitary hygiene napkins. Mater. Today Commun..

[83] Liu L., Zhang Y., Li C., Cao J., He E., Wu X., Wang F., Wang L. (2020). Facile preparation PCL/modified nano ZnO organic-inorganic composite and its application in antibacterial materials. J. Polym. Res..

[84] Abouelnaga A.M., El Nahrawy A.M. (2024). Spectroscopic investigation, dielectric and antimicrobial properties of chitin-cellulose@ZnO/CuO conductive nanocomposites. Spectrochim. Acta Part A Mol. Biomol. Spectrosc..

[85] Bai X., Lin C., Wang Y., Ma J., Wang X., Yao X., Tang B. (2020). Preparation of Zn doped mesoporous silica nanoparticles (Zn-MSNs) for the improvement of mechanical and antibacterial properties of dental resin composites. Dent. Mater..

[86] Toh-ae P., Lee-Nip R., Nakaramontri Y. (2023). Releasing of zinc ions from modified zinc oxide surfaces for improvement chemical crosslinks and antibacterial properties of acrylonitrile butadiene rubber films. Express Polym. Lett..

[87] Abdelghany A.M., Elamin N.Y., Younis S., Ayaad D.M. (2024). Polyvinyl pyrrolidone/carboxymethyl cellulose (PVP/CMC) polymer composites containing CuO nanoparticles synthesized via laser ablation in liquids. J. Mol. Liq..

[88] Fahmy T., Sarhan A. (2022). Investigation of optical properties and antibacterial activity of chitosan copper nanoparticle composites. Mater. Technol..

[89] Glazkova E., Bakina O., Rodkevich N., Mosunov A., Evstigneev M., Evstigneev V., Klimenko V., Lerner M. (2022). Antibacterial Properties of PMMA Functionalized with CuFe2O4/Cu2O/CuO Nanoparticles. Coatings.

[90] Shah B.A., Sardar A., Peng W., Din S.T.U., Hamayoun S., Li S., Yuan B. (2023). Photoresponsive CuS@polyaniline nanocomposites: An excellent synthetic bactericide against several multidrug-resistant pathogenic strains. Inorg. Chem. Front..

[91] Jardón-Maximino N., Cadenas-Pliego G., Ávila-Orta C.A., Comparán-Padilla V.E., Lugo-Uribe L.E., Pérez-Alvarez M., Tavizón S.F., Santillán G.d.J.S. (2021). Antimicrobial Property of Polypropylene Composites and Functionalized Copper Nanoparticles. Polymers.

[92] Govindasamy G.A., Sreekantan S., Saharudin K.A., Poliah R., Ong M.T., Thavamany P.J., Sahgal G., Tan A.A. (2024). Composition-Dependent Physicochemical and Bactericidal Properties of Dual Cu-TiO2 Nanoparticles Incorporated in Polypropylene. BioNanoScience.

[93] Karbowniczek J.E., Berniak K., Knapczyk-Korczak J., Williams G., Bryant J.A., Nikoi N.D., Banzhaf M., de Cogan F., Stachewicz U. (2023). Strategies of nanoparticles integration in polymer fibers to achieve antibacterial effect and enhance cell proliferation with collagen production in tissue engineering scaffolds. J. Colloid Interface Sci..

[94] Sebak M.A., Qahtan T.F., Asnag G.M., Abdallah E.M. (2022). The Role of TiO2 Nanoparticles in the Structural, Thermal and Electrical Properties and Antibacterial Activity of PEO/PVP Blend for Energy Storage and Antimicrobial Application. J. Inorg. Organomet. Polym. Mater..

[95] Balan R., Gayathri V. (2021). In-vitro and antibacterial activities of novel POT/TiO2/PCL composites for tissue engineering and biomedical applications. Polym. Bull..

[96] Du Y., Chen M., Wang B., Chai Y., Wang L., Li N., Zhang Y., Liu Z., Guo C., Jiang X. (2024). TiO2/Polystyrene Nanocomposite Antibacterial Material as a Hemoperfusion Adsorbent for Efficient Bilirubin Removal and Prevention of Bacterial Infection. ACS Biomater. Sci. Eng..

[97] Babers N., El-Sherbiny M.G.D., El-Shazly M., Kamel B.M. (2023). Mechanical and antibacterial properties of hybrid polymers composite reinforcement for biomedical applications. J. Biomater. Sci. Polym. Ed..

[98] Shaalan M., Vykydalová A., Švajdlenková H., Kroneková Z., Marković Z.M., Kováčová M., Špitálský Z. (2024). Antibacterial activity of 3D printed thermoplastic elastomers doped with carbon quantum dots for biomedical applications. Polym. Bull..

[99] Joy A., Megha M., Mohan C.C., Thomas J., Bhat S.G., Muthuswamy S. (2024). Novel polycaprolactone-based biomimetic grafts enriched with graphene oxide and cerium oxide: Exploring improved osteogenic potential. Mater. Today Chem..

[100] Kafali M., Şahinoğlu O.B., Tufan Y., Orsel Z.C., Aygun E., Alyuz B., Saritas E.U., Erdem E.Y., Ercan B. (2023). Antibacterial properties and osteoblast interactions of microfluidically synthesized chitosan–SPION composite nanoparticles. J. Biomed. Mater. Res. Part A.

[101] Sadeghi-Ghadi Z., Behjou N., Ebrahimnejad P., Mahkam M., Goli H.R., Lam M., Nokhodchi A. (2022). Improving Antibacterial Efficiency of Curcumin in Magnetic Polymeric Nanocomposites. J. Pharm. Innov..

[102] Guo W., Zhou B., Zou Y., Lu X. (2023). 4D Printed Poly(l-lactide)/(FeCl3–TA/MgO) Composite Scaffolds with Near-Infrared Light-Induced Shape-Memory Effect and Antibacterial Properties. Adv. Eng. Mater..

[103] Ahangaran F., Navarchian A.H. (2022). Towards the development of self-healing and antibacterial dental nanocomposites via incorporation of novel acrylic microcapsules. Dent. Mater..

[104] Yang Y., Xu Z., Guo Y., Zhang H., Qiu Y., Li J., Ma D., Li Z., Zhen P., Liu B. (2021). Novel core–shell CHX/ACP nanoparticles effectively improve the mechanical, antibacterial and remineralized properties of the dental resin composite. Dent. Mater..

[105] Yao S., Qin L., Wang Z., Zhu L., Zhou C., Wu J. (2022). Novel nanoparticle-modified multifunctional microcapsules with self-healing and antibacterial activities for dental applications. Dent. Mater..

[106] Wang Y., Wu Z., Wang T., Tang W., Li T., Xu H., Sun H., Lin Y., Tonin B.S.H., Ye Z. (2023). Bioactive Dental Resin Composites with MgO Nanoparticles. ACS Biomater. Sci. Eng..

[107] Benedini L., Laiuppa J., Santillán G., Baldini M., Messina P. (2020). Antibacterial alginate/nano-hydroxyapatite composites for bone tissue engineering: Assessment of their bioactivity, biocompatibility, and antibacterial activity. Mater. Sci. Eng. C.

[108] Ahmed S., Hussain R., Khan A., Batool S.A., Mughal A., Nawaz M.H., Irfan M., Wadood A., Avcu E., Rehman M.A.U. (2023). 3D Printing Assisted Fabrication of Copper–Silver Mesoporous Bioactive Glass Nanoparticles Reinforced Sodium Alginate/Poly(vinyl alcohol) Based Composite Scaffolds: Designed for Skin Tissue Engineering. ACS Appl. Bio. Mater..

[109] Shalini A., Deepa K., Meenakshi S., Al-Ansari M.M., Alhumaid L., Rajendran K., Dixit S., Lo H.M. (2024). A study on antibacterial and anti-inflammatory activity of xylitol-based polymeric nano-bioactive glass nanocomposites. Polym. Adv. Technol..

[110] Astashev M.E., Sarimov R.M., Serov D.A., Matveeva T.A., Simakin A.V., Ignatenko D.N., Burmistrov D.E., Smirnova V.V., Kurilov A.D., Mashchenko V.I. (2022). Antibacterial behavior of organosilicon composite with nano aluminum oxide without influencing animal cells. React. Funct. Polym..

[111] Bashal A.H., Khalil K.D., Abu-Dief A.M., El-Atawy M.A. (2023). Cobalt oxide-chitosan based nanocomposites: Synthesis, characterization and their potential pharmaceutical applications. Int. J. Biol. Macromol..

[112] Chacko S.K., Balakrishnan R., Kalarikkal N., Thomas N.G. (2024). Ternary Fiber Mats of PVDF-HFP/Cellulose/LiFe5O8 Nanoparticles with Enhanced Electric, Magnetoelectric, and Antibacterial Properties: A Promising Approach for Magnetic and Electric Field-Responsive Antibacterial Coatings. ACS Appl. Polym. Mater..

[113] Raja T., Al-Otibi F.O., Alharbi R.I., Mohanavel V., Velmurugan P., Karthikeyan S., Perumal M., Basavegowda N. (2023). A novel study of biological and structural analysis on Cissus quadrangularis fiber-reinforced CaO particulates epoxy composite for biomedical application. J. Mater. Res. Technol..

[114] Silva C., Bobillier F., Canales D., Antonella Sepúlveda F., Cament A., Amigo N., Rivas L.M., Ulloa M.T., Reyes P., Ortiz J.A. (2020). Mechanical and Antimicrobial Polyethylene Composites with CaO Nanoparticles. Polymers.

[115] Sathiyaseelan A., Zhang X., Lin J., Wang M.-H. (2024). In situ, synthesis of chitosan fabricated tellurium nanoparticles for improved antimicrobial and anticancer applications. Int. J. Biol. Macromol..

[116] Tamta A., Kumar R., Gouri V., Joshi R., Chandra B., Kandpal N.D. (2024). Synthesis, characterization and biological activities of NiO-cellulose nanocomposite. Curr. Chem. Lett..

